# Development and Evaluation of Machine Learning in Whole-Body Magnetic Resonance Imaging for Detecting Metastases in Patients With Lung or Colon Cancer: A Diagnostic Test Accuracy Study

**DOI:** 10.1097/RLI.0000000000000996

**Published:** 2023-06-26

**Authors:** Andrea G. Rockall, Xingfeng Li, Nicholas Johnson, Ioannis Lavdas, Shalini Santhakumaran, A. Toby Prevost, Shonit Punwani, Vicky Goh, Tara D. Barwick, Nishat Bharwani, Amandeep Sandhu, Harbir Sidhu, Andrew Plumb, James Burn, Aisling Fagan, Georg J. Wengert, Dow-Mu Koh, Krystyna Reczko, Qi Dou, Jane Warwick, Xinxue Liu, Christina Messiou, Nina Tunariu, Peter Boavida, Neil Soneji, Edward W. Johnston, Christian Kelly-Morland, Katja N. De Paepe, Heminder Sokhi, Kathryn Wallitt, Amish Lakhani, James Russell, Miriam Salib, Sarah Vinnicombe, Adam Haq, Eric O. Aboagye, Stuart Taylor, Ben Glocker

**Affiliations:** From the Department of Surgery and Cancer, Faculty of Medicine, Imperial College London, London, United Kingdom (A.G.R., X.L., I.L., T.D.B., N.B., G.J.W., N.S., A.L., A.H., E.O.A.); Imaging Department, Imperial College Healthcare NHS Trust, London, United Kingdom (A.G.R., T.D.B., N.B., A.S., J.B., A.F., N.S., K.W., A.L., J.R., M.S.); Imperial Clinical Trials Unit, Imperial College London, London, United Kingdom (N.J., S.S., A.T.P., J.W., X.L.); Centre for Medical Imaging, University College London, London, United Kingdom (S.P., H.S., A.P., S.T.); Department of Radiology, University College London Hospital, London, United Kingdom (S.P., H.S., A.P., S.T.); Cancer Imaging, School of Biomedical Engineering & Imaging Sciences, King's College London and Department of Radiology, Guy's & St Thomas' Hospitals NHS Foundation Trust, London, United Kingdom (V.G., C.K.-M.); Department of Biomedical Imaging and Image-Guided Therapy, Medical University of Vienna, Vienna General Hospital, Vienna, Austria (G.J.W.); Royal Marsden NHS Foundation Trust and The Institute of Cancer Research, London, United Kingdom (D.-M.K., C.M., N.T., N.S., C.K.-M., K.N.D.P.); Cancer Research UK and University College London Cancer Trials Unit, London, United Kingdom (K.R.); Faculty of Engineering, Department of Computing, Imperial College London, London, United Kingdom (Q.D., B.G.); King's Cancer Prevention Group, School of Cancer and Pharmaceutical Sciences, King's College London, London, United Kingdom (J.W.); Department of Radiology, Homerton NHS Foundation Trust, London, United Kingdom (P.B.); Paul Strickland Scanner Centre, Mount Vernon Hospital (H.S.); Department of Radiology, The Hillingdon Hospitals NHS Foundation Trust, London, United Kingdom (H.S.); Thirlestaine Breast Centre, Gloucestershire Hospitals NHS Foundation Trust, London, United Kingdom (S.V.); and Nightingale-Saunders Clinical Trials & Epidemiology Unit, King's Clinical Trials Unit, London, United Kingdom (A.T.P.).

**Keywords:** whole-body MRI, diffusion-weighted imaging, T2-weighted imaging, cancer staging, machine learning, MRI segmentation, metastasis detection, diagnostic test performance, human-in-the-loop, radiology read-time

## Abstract

**Objectives:**

Whole-body magnetic resonance imaging (WB-MRI) has been demonstrated to be efficient and cost-effective for cancer staging. The study aim was to develop a machine learning (ML) algorithm to improve radiologists' sensitivity and specificity for metastasis detection and reduce reading times.

**Materials and Methods:**

A retrospective analysis of 438 prospectively collected WB-MRI scans from multicenter Streamline studies (February 2013–September 2016) was undertaken. Disease sites were manually labeled using Streamline reference standard. Whole-body MRI scans were randomly allocated to training and testing sets. A model for malignant lesion detection was developed based on convolutional neural networks and a 2-stage training strategy. The final algorithm generated lesion probability heat maps. Using a concurrent reader paradigm, 25 radiologists (18 experienced, 7 inexperienced in WB-/MRI) were randomly allocated WB-MRI scans with or without ML support to detect malignant lesions over 2 or 3 reading rounds. Reads were undertaken in the setting of a diagnostic radiology reading room between November 2019 and March 2020. Reading times were recorded by a scribe. Prespecified analysis included sensitivity, specificity, interobserver agreement, and reading time of radiology readers to detect metastases with or without ML support. Reader performance for detection of the primary tumor was also evaluated.

**Results:**

Four hundred thirty-three evaluable WB-MRI scans were allocated to algorithm training (245) or radiology testing (50 patients with metastases, from primary 117 colon [n = 117] or lung [n = 71] cancer). Among a total 562 reads by experienced radiologists over 2 reading rounds, per-patient specificity was 86.2% (ML) and 87.7% (non-ML) (−1.5% difference; 95% confidence interval [CI], −6.4%, 3.5%; *P* = 0.39). Sensitivity was 66.0% (ML) and 70.0% (non-ML) (−4.0% difference; 95% CI, −13.5%, 5.5%; *P* = 0.344). Among 161 reads by inexperienced readers, per-patient specificity in both groups was 76.3% (0% difference; 95% CI, −15.0%, 15.0%; *P* = 0.613), with sensitivity of 73.3% (ML) and 60.0% (non-ML) (13.3% difference; 95% CI, −7.9%, 34.5%; *P* = 0.313). Per-site specificity was high (>90%) for all metastatic sites and experience levels. There was high sensitivity for the detection of primary tumors (lung cancer detection rate of 98.6% with and without ML [0.0% difference; 95% CI, −2.0%, 2.0%; *P* = 1.00], colon cancer detection rate of 89.0% with and 90.6% without ML [−1.7% difference; 95% CI, −5.6%, 2.2%; *P* = 0.65]). When combining all reads from rounds 1 and 2, reading times fell by 6.2% (95% CI, −22.8%, 10.0%) when using ML. Round 2 read-times fell by 32% (95% CI, 20.8%, 42.8%) compared with round 1. Within round 2, there was a significant decrease in read-time when using ML support, estimated as 286 seconds (or 11%) quicker (*P* = 0.0281), using regression analysis to account for reader experience, read round, and tumor type. Interobserver variance suggests moderate agreement, Cohen κ = 0.64; 95% CI, 0.47, 0.81 (with ML), and Cohen κ = 0.66; 95% CI, 0.47, 0.81 (without ML).

**Conclusions:**

There was no evidence of a significant difference in per-patient sensitivity and specificity for detecting metastases or the primary tumor using concurrent ML compared with standard WB-MRI. Radiology read-times with or without ML support fell for round 2 reads compared with round 1, suggesting that readers familiarized themselves with the study reading method. During the second reading round, there was a significant reduction in reading time when using ML support.

Patients being investigated for suspected or confirmed cancer often undergo multiple imaging tests to ascertain the initial TNM stage before formulating the final treatment strategy. The multicenter prospective NIHR Streamline studies compared the diagnostic accuracy of whole-body magnetic resonance imaging (WB-MRI) with standard staging pathways (CT ± regional MRI [rectum, liver, brain] ± FDG PET/CT), for initial staging in patients with newly diagnosed non–small cell lung or colorectal cancer.^1,2^ The studies also evaluated the number of tests required and the time taken before reaching the final treatment plan. The Streamline studies found that WB-MRI is a viable alternative to standard pathways with similar accuracy but reduced staging time and cost.^1,2^ However, WB-MRI has not yet been widely translated into staging pathways in lung and colon cancer, although there is more widespread use for staging bone disease in myeloma and to some extent in prostate and breast cancer.^3–5^ One speculative reason for this could be due to a perceived need for specialist expertise in reading WB-MRI, as well as the time taken to report the scans, due to the challenges of integrating a large number of complex sequences available on WB-MRI.

Significant developments in machine learning (ML), especially deep learning (DL), have opened the possibility of automated segmentation and lesion detection on CT and MRI.^6–11^ However, ML techniques for cancer lesion detection on WB-MRI have not been widely researched.

The aim of this study was to develop and clinically test an algorithm for cancer lesion detection on WB-MRI scans in patients recruited to the Streamline studies with lung and colorectal cancer, using a human-in-the-loop approach. The intended use of the algorithm was a concurrent ML heat map on WB-MRI images at the time of interpretation, alerting radiologists to potential metastases, with the hypothesis that ML support would improve lesion detection and reduce read-times. Secondary objectives included evaluating reader performance for inexperienced WB-MRI readers and detection of the primary tumor with or without ML.

## MATERIALS AND METHODS

### Study Design

This retrospective study obtained ethical approval (ICREC 15IC2647, ISRCTN 23068310). Patients gave written consent in Streamline studies (ISRCTN43958015 and ISRCTN50436483) for use of deidentified data for future research. TNM stages for each case were provided by the source study.

Scans were acquired at 16 recruitment sites between February 2013 and September 2016, using a minimum WB-MRI protocol.^12^ The Streamline consensus reference standard for sites of disease was used.^1,2^ In brief, this consisted of multidisciplinary consensus meetings, which retrospectively considered all imaging, treatment interventions, histopathology, and patient outcomes for at least 12 months after cancer diagnosis to ascertain the cancer stage and site of metastasis at diagnosis.

Cases were randomly allocated to model training and clinical testing (stratified by primary tumor type [lung or colon], presence of at least 1 metastatic lesion, and recruitment site), ensuring sufficient cases with and without metastases were allocated to the testing set to meet the power calculation, with all other cases allocated to training.

### Data Preparation

All DICOM data received were initially included. Individual anatomical imaging stations of the 3 key sequences (defined as axial T2-weighted [T2WI], diffusion-weighted [DWI], or apparent diffusion coefficient [ADC] map) were stitched into a single DICOM stack and converted to NIfTI (https://nifti.nimh.nih.gov/).^11,13^ Cases were excluded due to absence of a key sequence or technical failure (NIfTI conversion or running the algorithm).

All visible disease sites (primary tumor and metastases) were segmented by trained radiologists using ITK-Snap, on T2WI and DWI NIfTI volumes based on the location, size, and number of lesions identified by the Streamline trials reference standard.^14^ Not all sites could be identified on the WB-MRI, as the source reference standard included metastases that subsequently became radiologically visible within 6 months, considering them likely present (although occult) at initial staging (see Supplemental Digital Content 1, http://links.lww.com/RLI/A825, which shows the visible sites for ground truth segmentation against the reference standard).

#### Data Availability

**FIGURE 1:**
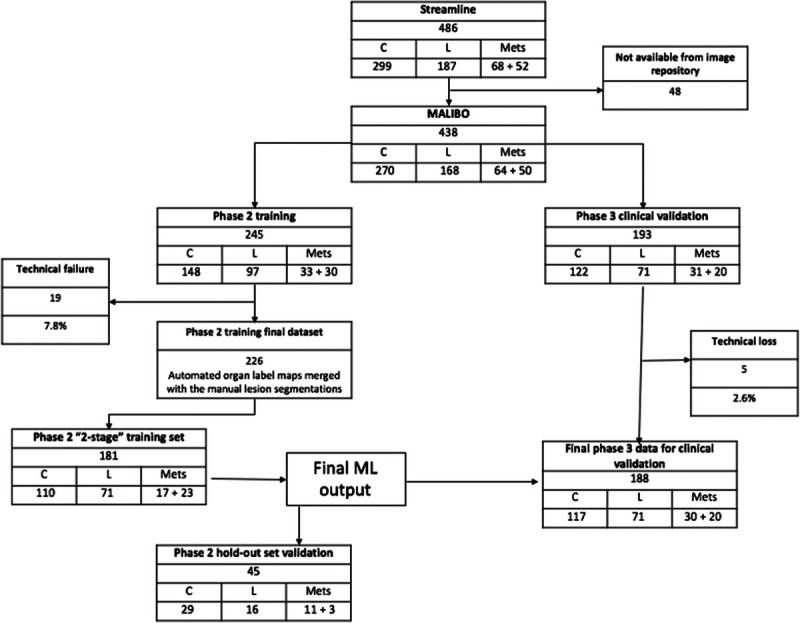
CONSORT diagram demonstrating distribution of cases to phase 2 training, with internal validation data set and phase 3 clinical test data set. C = colon cancer, L = Lung cancer.

Among 486 patients in the Streamline studies, 438 WB-MRI scans were available for the study (270 colorectal and 168 lung cancer, 114 with metastases) (Fig. 1). The stages of disease are provided in Table 1.

**TABLE 1:** Stage Distribution of the Cases Allocated to Radiology Reads (Test Set, n = 188)

	Number	%
Colon cancer stage	n = 117	
T1	7	6
T2	25	21
T3	69	59
T4	16	14
N0	55	47
N1	35	30
N2	27	23
M0	87	74
M1	30	26
Lung cancer stage	n = 71	
T1a	10	14
T1b	9	13
T2a	18	25
T2b	8	11
T3	14	20
T4	12	17
N0	42	59
N1	10	14
N2	13	18
N3	6	8
M0	51	72
M1	20	28

Among 245 scans allocated to training, there were 19 technical failures with 226 evaluable for training (n = 181) and internal validation (n = 45). Among 193 scans allocated to the test set, there were 5 technical failures (missing ADC n = 2, corrupted DWI n = 1, failure of NIFTI conversion and upload n = 2), leaving 188 evaluable scans (117 colon, 71 lung, 50 cases with metastases).

### Machine Learning Model

**FIGURE 2:**
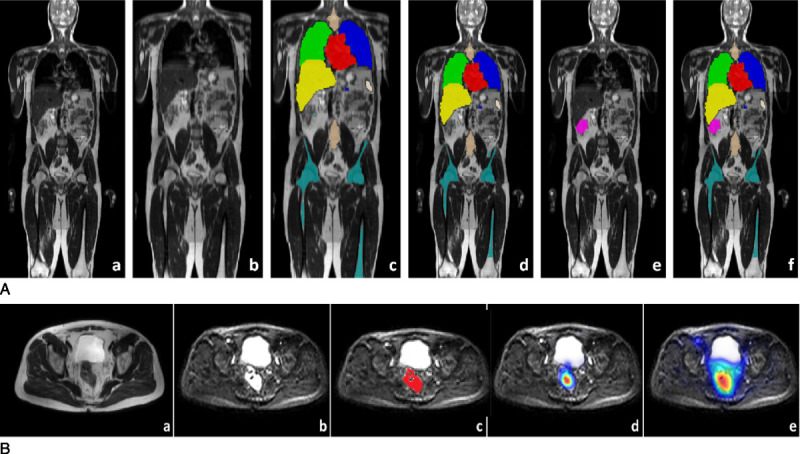
Data generation process for the 2-stage model training approach. Panel A: A, An example of a T2WI WB-MRI scans from a participant in training set. B, After registration to a template scan from the healthy volunteer study. C, Output of the organ. Segmentation algorithm developed in healthy volunteer study. D, After mapping the organ segmentations back to the original scan from training data. E, Manual lesion segmentation overlaid on the T2WI scan. F, Merged organ segmentations and cancer lesion segmentation overlaid on the T2WI scan, which is used for training the final multiclass segmentation algorithm. Panel B: Cancer lesion detection training. A, Input T2WI scan (different patient to panel A). B, Diffusion-weighted scan. C, Manual lesion segmentation (based on reference standard) from T2WI image overlaid on diffusion scan. D, Postprocessed lesion probability map from the convolutional neural network (CNN) algorithm (deep medic). E, Postprocessed lesion probability map from the classification forest (CF) algorithm.

We investigated several ML algorithms and different training strategies for the task of malignant lesion segmentation in multichannel WB-MRI. Details about the tested alternatives can be found in the Supplemental Digital Content 2, http://links.lww.com/RLI/A826. The final ML model was based on deep convolutional neural networks (CNNs), developed with a 2-stage strategy. We first leveraged an existing CNN algorithm for the segmentation of healthy organs, developed in a previous healthy volunteer study.^11^ Running this multiorgan CNN segmentation algorithm on all the training data provided automatic organ maps for all patient scans. This required an intermediate step of registering phase 2 data with a rigid registration algorithm to a template subject from the healthy volunteer data (Fig. 2). This was to compensate for the different fields of view of the healthy volunteer study and the current patient study. Although the healthy volunteer WB-MRI data covered the body from shoulders to knees, the patient study data included the head, which affects the performance of the organ segmentation algorithm. The registration was automatic and fast, and was only required in order to obtain the organ masks. The organ masks were then mapped back with the inverse transformation to the original patient training data. For the patient training data set, there was no reference segmentation of organs to compare with, so we assessed the quality of these segmentations visually, and they appeared to be sufficient for the second stage.

The automatically generated organ maps were then merged with the manually segmented primary and metastatic malignant lesions on T2WI and DWI training data (Fig. 2). This resulted in all scans allocated to training having multiclass segmentation maps where the organ labels were generated automatically using the previously developed CNN algorithm, although the cancer lesions were labeled manually. We then used the training set for training a CNN for joint organ and lesion segmentation, using the DeepMedic architecture.^15^ This CNN model was then capable of predicting jointly the organ labels and malignant lesions on new unseen test data. The generated probability heat maps for the lesion class were postprocessed by applying Gaussian smoothing with a kernel size of 5 mm, normalized to the range [0, 1], and thresholded to reduce false-positive predictions. Parameters for the postprocessing were selected based on visual assessment of the 45 internal validation cases. The final lesion probability heat maps were converted to DICOM and uploaded to PACS to enable overlay with the original WB-MRI scans. Each WB-MRI scan was copied (one with and one without ML heat map series) to allow masking.

### Radiology Reads

Eighteen experienced readers were Streamline radiologists (n = 7) or those routinely reporting WB-MRI for tumor boards (n = 11). Seven inexperienced readers included consultants who do not read WB-MRI (n = 3) and board-certified senior radiology trainees (n = 4). All readers were trained to use the Biotronics3D reading platform, including optional superimposition of ML heat maps (see Supplemental Digital Content 3, http://links.lww.com/RLI/A827, which shows the reader training manual with appearance of the PACS reading setup with heat maps).

**FIGURE 3:**
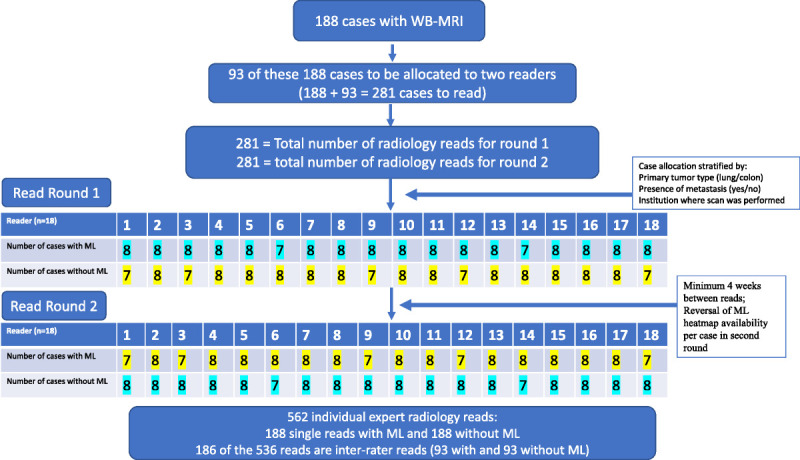
Flowchart for blinded sequential reads methodology for 18 experienced readers. Final test set of 188 reads together with randomly selected 93 cases to be read by 2 radiologists provided a total of 281 reads for each of 2 reading rounds. Stratification of cases was performed to ensure a reasonable equivalence of cases with lung and colon cancer, with or without metastases and by institution, to ensure that readers had a range of cases and that a reader did not get allocated cases from their own institution. A mixture of cases with and without machine learning (ML) support was available at each reading round in order to avoid training bias. A minimum of 4 weeks was scheduled between reading rounds to reduce recall bias.

Cases in the test set (n = 188) were randomly allocated to readers, stratified by tumor type (colon or lung), presence of at least 1 metastasis or none, and recruitment site from the Streamline study to ensure readers had a similar set of reads, but not from their own institution. In addition, 93 randomly selected cases were allocated to be read by a second experienced reader, in order to evaluate interrater agreement (see Fig. 3 for the reading flow diagram). Readers were blinded as to which cases were allocated for interrater agreement. Each of 18 experienced readers therefore had 15 to 16 allocated cases, which they read twice over 2 reading rounds, separated by a minimum of 4 weeks to reduce recall bias.

In a similar method, 7 inexperienced readers were allocated either 10 or 14 reads per read round, based on capacity of the reader, 4 of which were included to evaluate interrater agreement.

To prevent any training bias, the case order (with or without ML support for read round 1, then reversed for read 2) was balanced to allow equivalent number of cases with ML in each round (Fig. 3).

Intrarater assessments performed by any readers available for a third reading round were randomly allocated 6 colon and 4 lung cases selected from their original allocation and then assigned with or without ML (1:1).

Radiologists reporting their findings to a trained scribe who filled case report forms, including detailed identification of the primary tumor, metastatic sites, and staging questions for readers (see Supplemental Digital Content 4, http://links.lww.com/RLI/A828, which provides the case report forms used for the data capture). The reader could choose to use or ignore the ML heat map (if available) to inform their opinion. Read-time was recorded from time images were loaded on screen to completion of the diagnostic read.

#### Statistical Analysis

Sample size for detecting a significant difference (superiority) between ML and non-ML in the primary outcome (per-patient sensitivity) required 141 patients without and 51 with metastases (see Supplemental Digital Content 5, http://links.lww.com/RLI/A829, which provides the full statistical analysis plan, including power calculation). Two-sided McNemar test for paired proportions, with 95% confidence intervals (CIs), was used to measure differences in sensitivity and specificity between reads with or without ML. Significance testing was based on the binomial distribution of the discordant pairs with results deemed statistically significant at *P* < 0.05. Regression analysis was used to investigate the paired difference in read-time based on ML usage. Fixed effects for read type (colon/lung) and read round of ML assistance (1/2) were included alongside a clustering effect for reader experience. SAS v9.4 (SAS Institute Inc, Cary, NC) was used for all analyses.

## RESULTS

### Results of Model Training

#### Results for Machine Learning Model Development

The results for the comparison of different ML models and training strategies are provided in the Supplemental Digital Content 2, http://links.lww.com/RLI/A826. The final model that was used by the readers was selected based on a quantitative analysis of voxel-wise lesion segmentation performance and using visual assessment on the 45 internal validation cases. When applied to the clinical test set, 70% of ground truth segmented cancer lesions had recall scores above 50%, meaning that, in 70% of malignant lesions, at least 50% of the voxels were considered to be malignant by the algorithm. It is important to note that precision and recall in the validation set (see Supplemental Digital Content 2, http://links.lww.com/RLI/A826) are on a voxel-level, whereas in the human-in-the-loop reader study, sensitivity and specificity were evaluated on a lesion-level.

### Results of Reader Performance

#### Per-Patient Sensitivity and Specificity for Detection of Metastatic Disease

##### Experienced Readers

Among a total of 562 reads (281 with and 281 without ML support) by experienced radiologists, 186 (93 with and 93 without ML) were read by 2 experienced radiologists. The sensitivity and specificity for identifying patients with and without metastatic disease, according to reader experience, is shown in Table 2.

**TABLE 2:** Detection of Metastases by Radiologists Reading With or Without ML Support Against Reference Standard

		All Reference Standard Positive, n = 50		All Reference Standard Negative, n = 138			
	n	TP	FN	TN	FP	Specificity %	Sensitivity %
Experienced readers	18						
Reads without ML support	188	35	15	121	17	87.7	70.0
Reads with ML support	188	33	17	119	19	86.2	66.0
Difference in proportions						−1.5 (95% CI, −6.4, 3.5; *P* = 0.387)	−4.0 (95% CI, −13.5, 5.5; *P* = 0.344)
		Subset Reference Standard Positive, n = 15		Subset Reference Standard Negative, n = 38			
Inexperienced readers	7						
Reads without ML support	53	9	6	29	9	76.3	60.0
Reads with ML support	53	11	4	29	9	76.3	73.3
Difference in proportions						0.0 (95% CI, −15.0, 15.0; *P* = 0.613)	13.3 (95% CI, −7.9, 34.5; *P* = 0.313)

Per-patient sensitivity and specificity for experienced and inexperienced WB-MRI readers.

ML, machine learning.

Of the 138 patients without metastatic disease, readers correctly identified 119 (specificity, 86.2%) and 121 (specificity, 87.7%), with and without ML, respectively, a difference in proportions of −1.5% (95% CI, −6.4%, 3.5%; *P* = 0.39). It should be noted that, in expecting 141 cases originally, the loss of power from the missing data is marginal (0.894).

Of the 50 patients with metastatic disease, readers correctly identified 33 (sensitivity, 66.0%) and 35 (sensitivity, 70.0%) with and without ML, a difference of −4.0% (95% CI, −13.5%, 5.5%; *P* = 0.34).

##### Inexperienced Readers

Among a total of 161 reads by inexperienced radiologists, 56 were read by 2 inexperienced radiologists. For inexperienced readers, per-patient specificity was 76.3% (29 of 38) with or without ML, a difference of 0% (95% CI, −15.0%, 15.0%; *P* = 0.613), with sensitivity of 73.3% (11 of 15) and 60.0% (9 of 15), respectively, a difference of 13.3% (95% CI, −7.9%, 34.5%; *P* = 0.313).

#### Per-Site Sensitivity and Specificity for Detection of Metastatic Disease

A breakdown of the specificity and sensitivity rates per site of lesion can be found in Table 3 for experienced readers. Specificity was not affected based on usage of the ML algorithm with the difference in proportions ranging from 1.6% down to −0.5%. In all cases, per-site specificity remained above 95%.

**TABLE 3:** Sensitivity and Specificity of Detection of Metastases by Radiologists Reading With or Without ML Support

	Specificity			Difference in Proportions			Sensitivity			Difference in Proportions		
Site	n	ML	No ML	∆	LCI	UCI	n	ML	No ML	∆	LCI	UCI
Liver	165	98.2%	98.2%	0.0%	−2.4	2.4	23	60.9%	69.6%	−8.7%	−20.2	2.8
Lung	178	95.5%	95.5%	0.0%	−2.7	2.7	10	10.0%	0.0%	10.0%	−8.6	28.6
Adrenal	184	98.4%	96.7%	1.6%	−0.2	3.5	4	50.0%	50.0%	0.0%	0.0	0.0
Kidney	187	100.0%	100.0%	0.0%	0.0	0.0	1	0.0%	0.0%	0.0%	0.0	0.0
Brain	182	98.9%	98.9%	0.0%	0.0	0.0	6	66.7%	50.0%	16.7%	−13.2	46.5
Pleura	187	97.3%	97.9%	−0.5%	−2.9	1.8	1	0.0%	0.0%	0.0%	0.0	0.0
Spleen	188	100.0%	100.0%	0.0%	0.0	0.0	NA					
Pancreas	188	100.0%	100.0%	0.0%	0.0	0.0	NA					
Peritoneum	185	97.8%	98.4%	−0.5%	−1.6	0.5	3	0.0%	33.3%	−33.3%	−86.7	20.0
Bowel	188	99.5%	99.5%	0.0%	−1.5	1.5	NA					
Chest	188	100.0%	100.0%	0.0%	0.0	0.0	NA					
Pelvis (nonskeletal)	186	99.5%	100.0%	−0.5%	−1.6	0.5	2	0.0%	0.0%	0.0%	0.0	0.0
Skull	187	100.0%	100.0%	0.0%	0.0	0.0	1	0.0%	0.0%	0.0%	0.0	0.0
Cervical spine	188	100.0%	100.0%	0.0%	0.0	0.0	NA					
Thoracic spine	184	99.5%	100.0%	−0.5%	−1.6	0.5	4	25.0%	0.0%	25.0%	−17.4	67.4
Lumbar spine	184	99.5%	98.9%	0.5%	−0.5	1.6	4	25.0%	0.0%	25.0%	−17.4	67.4
Sternum	187	100.0%	100.0%	0.0%	0.0	0.0	1	100.0%	100.0%	0.0%	0.0	0.0
Pelvis (skeletal)	186	99.5%	100.0%	−0.5%	−1.6	0.5	2	0.0%	50.0%	−50.0%	−119	19.3
Clavicle	NA						NA					
Ribs	188	100.0%	100.0%	0.0%	0.0	0.0	NA					
Other skeletal	188	100.0%	100.0%	0.0%	0.0	0.0	NA					

Per-site, for experienced WB-MRI readers.

ML, machine learning.

Investigating per-site sensitivity was hindered as only 2 sites (liver and lung) had 10 or more positive cases based on the reference standard. Liver produced a sensitivity difference of −8.7% (95% CI, −20.2, 2.8) in metastatic tumor detection when using ML while lung provided very low sensitivity rates with 10.0% (95% CI, 0.5, 45.9) in the ML arm and 0% (95% CI, 0.0, 34.5) without ML (Table 3). It should be noted that these intervals are wide due to small sample sizes.

Please refer to Supplemental Digital Content 6, http://links.lww.com/RLI/A830, for table of per-site sensitivity for inexperienced readers.

#### Per-Patient Sensitivity and Specificity for Detection of Primary Tumor

There was no significant difference in detection of the primary tumor with or without ML. Of 71 primary lung cancers (70 of which were visible for ground truth segmentation), 70 were detected by experienced readers with or without ML (sensitivity, 98.6%), a difference of 0.0% (95% CI, −2.0%, 2.0%; *P* = 1.00). Twenty of 20 lung tumors visible for ground truth segmentation were detected by inexperienced readers (sensitivity, 100%; difference, 0.0%; 95% CI, −0.0%, 0.0%; *P* = 1.00). Of 118 primary colon cancers, 116 were identified for ground truth segmentation. All 118 cases were read by experienced radiology readers, 105 were detected with ML support, and 107 were detected without ML, respectively (sensitivities of 89.0% and 90.6%; difference, −1.7%; 95% CI, −5.6%, 2.2%; *P* = 0.65). Of 33 primary colon cancers evaluated by inexperienced readers, 31 and 29 were detected with and without ML (sensitivities of 93.9% and 87.9%; difference, 6.1%; 95% CI, −1.0%, 13.1%; *P* = 0.39).

### Results of Time to Complete Reads

Combining rounds 1 and 2, the overall mean (SD) reading time for experienced readers with ML was 560 (260) seconds. The time increases to 595 (610) seconds without ML (Table 4), thus using ML, the unadjusted mean reading time fell by an average of 35 seconds (95% CI, −60, 140), an average percentage reduction of 6.2% (95% CI, −10.0%, 22.8%). Round 2 read-times were markedly lower regardless of ML assistance or read type, dropping from 689 (604) to 467 (226), an average of 222 seconds (95% CI, 129, 314) or 32.2% (95% CI, 18.7%, 45.6%).

**TABLE 4:** Reading Times Analysis: Mean (SD) and Median [IQR] Read-Time in Seconds by Arm (With or Without ML Support), Reader Experience, and Read Round—All Cases, Colon Cases, and Lung Cases

	Experienced Readers						Inexperienced Readers					
	Without ML			With ML			Without ML			With ML		
Read Round	n	Mean (SD)	Median [IQR]	n	Mean (SD)	Median [IQR]	n	Mean (SD)	Median [IQR]	n	Mean (SD)	Median [IQR]
All reads*	188	595 (610)	480 [300–720]	188	560 (260)	540 [360–720]	53	691 (412)	600 [420–900]	53	645 (329)	600 [360–840]
Round 1	92	715 (824)	600 [360–780]	96	663 (259)	600 [450–810]	26	842 (476)	630 [540–1020]	27	736 (382)	660 [360–840]
Round 2	96	481 (236)	420 [300–600]	92	453 (216)	390 [300–570]	27	544 (275)	540 [300–660]	26	552 (235)	480 [360–720]
Round 3	21	454 (206)	420 [300–540]	20	411 (156)	390 [300–510]	7	351 (112)	360 [240–480]	6	410 (158)	360 [300–420]

	Experienced Readers						Inexperienced Readers					
Colon	Without ML			With ML			Without ML			With ML		
Read Round	n	Mean (SD)	Median [IQR]	n	Mean (SD)	Median [IQR]	n	Mean (SD)	Median [IQR]	n	Mean (SD)	Median [IQR]
All reads*	117	597 (357)	540 [360–780]	117	560 (257)	540 [360–720]	33	758 (481)	600 [360–1020]	33	705 (368)	660 [360–840]
Round 1	58	703 (417)	600 [360–840]	59	655 (249)	600 [420–840]	13	1057 (559)	960 [600–1320]	20	756 (423)	600 [420–840]
Round 2	59	492 (249)	480 [300–600]	58	463 (228)	390 [300–660]	20	564 (303)	540 [300–780]	13	626 (259)	660 [420–720]
Round 3	12	455 (161)	450 [330–570]	10	432 (162)	420 [300–600]	5	396 (100)	420 [360–480]	3	460 (227)	360 [300–720]

	Experienced Readers						Inexperienced Readers					
Lung	Without ML			With ML			Without ML			With ML		
Read Round	n	Mean (SD)	Median [IQR]	n	Mean (SD)	Median (IQR)	n	Mean (SD)	Median [IQR]	n	Mean (SD)	Median [IQR]
All reads*	71	593 (885)	420 [300–720]	71	559 (268)	540 [360–720]	20	579 (229)	570 [450–630]	20	546 (228)	510 [360–660]
Round 1	34	736 (1253)	540 [360–720]	37	675 (276)	660 [480–780]	13	628 (243)	600 [480–660]	7	677 (249)	600 [540–840]
Round 2	37	462 (213)	420 [300–660]	34	434 (195)	390 [300–540]	7	489 (181)	540 [360–600]	13	475 (189)	420 [360–600]
Round 3	9	453 (265)	420 [300–480]	10	390 (156)	330 [300–420]	2	240 (0)	240 [240–240]	3	360 (60)	360 [300–420]

*Not including intrarater round 3 reads; ML, machine learning; SD, standard deviation; IQR, interquartile range.

Two read-times were recorded at over 2 hours, hence Table 4 also displays median interquartile range (IQR) read-times as well as additional subgroup output by reader ability (experienced/inexperienced), read order (round 1 and 2), and read type (colon and lung). On average, experienced readers completed their reads 100 seconds (95% CI, −75, 274) faster (or 12.6%; 95% CI, −9.6%, 34.8%) than their inexperienced counterparts for round 1 reads, and 81 seconds (95% CI, 10, 152) faster (or 14.8%; 95% CI, 1.2%, 27.8%) for round 2.

To investigate ML versus non-ML difference in read-time, a regression analysis was carried out using paired data comparing ML against their respective non-ML read. The regression model was adjusted for fixed-effect covariates: read tumor type (lung and colon) and read round (whether ML was used in the first or second round of reading). A clustering effect for reader experience was also included. Assumptions for regression modeling held and residuals were found to be normally distributed. Table 5 contains regression estimates (in seconds and as a percentage) for estimated effects of Read Round and Read Package when investigating paired ML versus non-ML difference in read-time. Although package type was not found to influence difference in read-time, the output indicated read round to have a significant (*P* = 0.0281) effect. The estimated effect on ML/non-ML difference between rounds 1 and 2 is −486 seconds (95% CI, −760, −213). Post hoc testing difference of least-square means to estimate the subsequent effect on read-time specifically when using ML at round 2 is −286 (95% CI, −370, −201) seconds. Similar post hoc testing of percentage difference estimated ML to reduce round 2 read-times by −11% (95% CI, −61%, 26%).

**TABLE 5:** Estimated Fixed Effects for Difference in Paired ML and Non-ML Reads From Regression Model in Seconds and as Percentage

	Effect	Value	Effect Estimate (95% CI)
Secs	Intercept		226 (−250, 702)
	ReadRound	Round 2	−486 (−760, −213)*
	Package	Colon	−51 (−626, 524)
%age	Intercept		64% (−32%, 160%)†
	ReadRound	Round 2	−77% (−113%, −41%)*
	Package	Colon	−9% (−43%, 25%)

Model investigating difference in ML and non-ML reading time in seconds and percentage adjusted for fixed effects Read round (when ML is applied, first or second round) and read package (lung or colon cancer). Additional clustering effect applied for reader experience.

Value estimates relate to the overall estimated effect adjusted for all other covariates within the model. Intercept refers to value at reference standards round 1 and lung packages.

*P < 0.05.

†*P* < 0.1.

## INTERREADER AND INTRAREADER ANALYSIS

Based on pairing 93 reads among 18 readers, Cohen κ for the interobserver variance among experienced readers with ML was 0.64 (95% CI, 0.47, 0.81). Without ML, the interobserver variance was unaffected with κ statistic of 0.66 (95% CI, 0.47, 0.81). This can be interpreted as moderate agreement between readers.

For intrarater reads, based on a sample of 30 tests, the corresponding κ statistics when comparing round 3 experienced reads with their counterpart in round 1 or 2 was 0.61 (95% CI, 0.13, 1.00) and 0.46 (95% CI, 0.10, 0.74) with and without ML, respectively, which are too imprecisely estimated in this current study sample.

## DISCUSSION

Whole-body MRI is an emerging imaging modality for staging lung and colon cancers, with similar accuracy to standard of care pathways, but with reduced staging time and cost.^1,2^ However, WB-MRI has not been widely translated into staging pathways. The perceived need for specialist expertise and the time needed to read WB-MRI may be a barrier to clinical translation.

We developed an algorithm to detect malignant lesions on WB-MRI, which generated heat maps for sites of lesions for review by radiologists, with the aims of improving detection of disease by radiologists and the speed of the radiology read. Machine learning support did not conclusively affect reader performance, with nonsignificant results when investigating differences in specificity (95% CI, −13.4%, 5.5%) and sensitivity (−6.4%, 3.5%) for detecting metastases. However, the data indicate that ML may not hinder the read process. When considering all reads from rounds 1 and 2, for all readers, reading time with ML had an average percentage reduction of 6.2% (95% CI, −10.8%, 22.8%). A 32% decrease in reading time between the first and second reading rounds is likely to indicate that radiologists took the first round to become familiarized with the reading environment on the cloud-based PACS, with the addition of ML heat maps, despite ensuring that each reader had been given prior training on the platform. Once familiarized with the reading environment, a potential benefit of ML in reading time may exist as round 2 reads were modeled to be 286 (95% CI, 201, 370) seconds (11%) faster with ML, when considering read round, reader experience and tumor type in regression analysis. It may be speculated that readers could more rapidly find lesions on the WB-MRI scan with ML heat map indicating sites to review, although higher detection rate was not achieved.

Radiology read-times overall were lower than anticipated, despite readers being allocated ample time. This may be due to reading with the scribe, who directed questions concerning particular aspects of the scan thereby creating an efficient reading system, particularly as the reader knew they were being timed. The stitched stacks for T2WI, DWI, and ADC may have meant that some other available sequences were not fully reviewed, as the available number of unprocessed sequences in many cases was very large. Unlike in the Streamline study, the generated scan report was not used for clinical decision-making, potentially reducing time spent on the read. The slightly shorter mean reading times using ML of approximately 6% overall would be unlikely to affect daily practice. All readers were inexperienced in reading with ML output in WB-MRI and the need to check appearances on the ML heat maps might have slowed down reading, particularly during the first round. However, ML output did not make overall reading times significantly longer. Once readers were familiarized with using the heat maps, there was a statistically significant drop in reading times by 11%, which could be meaningful clinically.

To our knowledge, no publication has investigated the diagnostic performance of human-ML radiology reading for detection of lung or colon cancer metastases in WB-MRI. Machine learning has been applied to WB-MRI in chronic nonbacterial osteitis, lymphoma, and characterization of myeloma bone lesions^9,16,17^ but with no ML-human-in-the-loop reader outputs.

Change in reading time when using ML or DL has been evaluated in other studies, with different imaging modalities and tumor types. A decrease in reading time of 11% has been reported in the detection of lung nodules, using DL.^18^ The use of DL support in breast tomosynthesis resulted in a mean decrease in reading time from 41 to 36 seconds, a mean decrease of 11%, with a range of reported decreases of between 14% and 52.7%.^19–21^ In prostate MRI, DL support reduced reading time by 21%, from a median of 103 to 81 seconds.^22^

Strengths of this study include the fact that WB-MRI scans were obtained from a prospective multicenter multivendor study, with multiparametric sequences. A relatively large number of independent radiologists took part in the study. The use of a scribe for CRF filling ensured homogeneity of reading methods, technical support to use the ML heat maps (a first for all the readers), and independent reading time measurement. The PACS cloud-based reading platform allowed blinded worklists, and all reads were done in a normal radiology reporting room, in an attempt at replicating daily work.

There were several challenges. Limited numbers of cases with metastatic lesions and the variety of scanners and slight differences in protocols all impacted on algorithm training. Because of time and resource constraints, it was not feasible to establish larger estimates of interrater and intrarater agreement. We did not attempt to train a model for automated disease diagnosis, but we focused on high sensitivity for lesion detection for review by radiologist. Performance for detection of metastases on a per-patient basis did not reach the high specificity of the Streamline studies (95% colon, 93% lung cohorts) but sensitivities were more similar (67% colon, 50% lung cohort).^1,2^ Speculative reasons for this could be that readers were allocated scans from a multitude of hospitals and vendors, with variable sequence appearances. Also, results were not being used for clinical care potentially changing reader behavior, whereas in Streamline, radiologists read WB-MRI from their site and results were used in treatment planning. In both the current and Streamline studies, the readers were blinded to other clinical data that may impact on diagnostic interpretation.

## CONCLUSIONS

A 2-step approach was developed to train a model for cancer lesion detection in WB-MRI of lung or colon cancer. Clinical validation by radiologists demonstrated equivalent performance for the detection of metastatic lesions on WB-MRI with or without ML support. There was a modest decrease in reading time when ML was used for later read rounds, although additional research is required to ascertain whether ML provides an overall time benefit. The study highlights the important impact of read round when analyzing read-time in the evaluation of ML applications.

## Supplementary Material
