# Supplementary material for: Development and Evaluation of Machine Learning in Whole-Body Magnetic Resonance Imaging for Detecting Metastases in Patients With Lung or Colon Cancer: A Diagnostic Test Accuracy Study
Source: Invest Radiol. 2023 Jun 26;58(12):823–31. doi: 10.1097/RLI.0000000000000996 (PMC10662596; doi:10.1097/RLI.0000000000000996)
Supplement: Supplementary file 1 [file ir-58-823-s001.docx]

Supplemental Digital Content 6

Supplementary Table S2.

Per-site sensitivity for inexperienced readers with and without ML

|  | | | |  |  |  |
| --- | --- | --- | --- | --- | --- | --- |
|  |  |  |  |  |  |  |
|  |  | Sensitivity | | Difference in Proportions | | |
| Site | n | ML | No ML | ∆ | LCI | UCI |
| liver | 7 | 71.40% | 71.40% | 0.00% | (0.0, | 0.0) |
| brain | 3 | 66.70% | 100.00% | -33.30% | (-78.1, | 11.4) |
| lung | 2 | 0.00% | 0.00% | 0.00% | (0.0, | 0.0) |
| adrenal | 1 | 100.00% | 0.00% | 100.00% | (100, | 100) |
| kidney | 1 | 0.00% | 0.00% | 0.00% | (0.0, | 0.0) |
| pleura | 1 | 0.00% | 0.00% | 0.00% | (0.0, | 0.0) |
| peritoneum | 1 | 0.00% | 0.00% | 0.00% | (0.0, | 0.0) |
