# Supplementary material for: Development and Evaluation of Machine Learning in Whole-Body Magnetic Resonance Imaging for Detecting Metastases in Patients With Lung or Colon Cancer: A Diagnostic Test Accuracy Study
Source: Invest Radiol. 2023 Jun 26;58(12):823–31. doi: 10.1097/RLI.0000000000000996 (PMC10662596; doi:10.1097/RLI.0000000000000996)
Supplement: Supplementary file 2 [file ir-58-823-s002.docx]

**Supplemental Digital Content 3.**

# Radiology reader training manual

# User manual for using 3D Biotronics platform

**User Manual for training radiologists to use 3D Biotronics platform**

Go to Google Chrome and open <https://www.3dnetmedical.com/portal>.

Login with your username and password (make sure this is the username that you have been given that links you to the MALIBO study folder), go then to **PACS** (upper row on the left side)


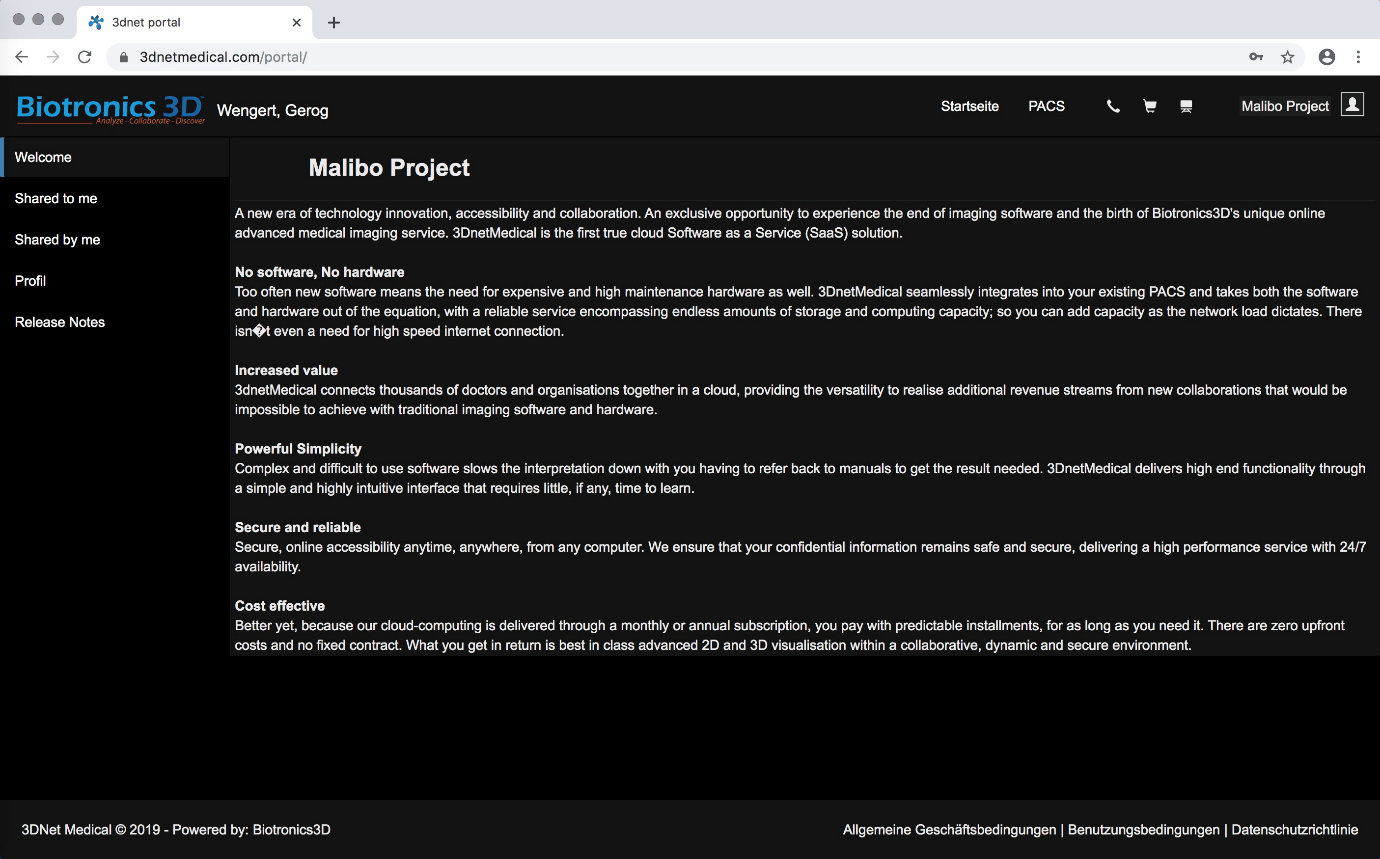


----------------------------------------------------------------------------------------------------------------

A window with 80 training cases will open

For individual sear, please use the **search bar**. We suggest:

**STL-039 -070, -103, -211**

**STC-007, -089 (though overly does not work on this one), -096, -274**

By double clicking a STL/STC case will open.


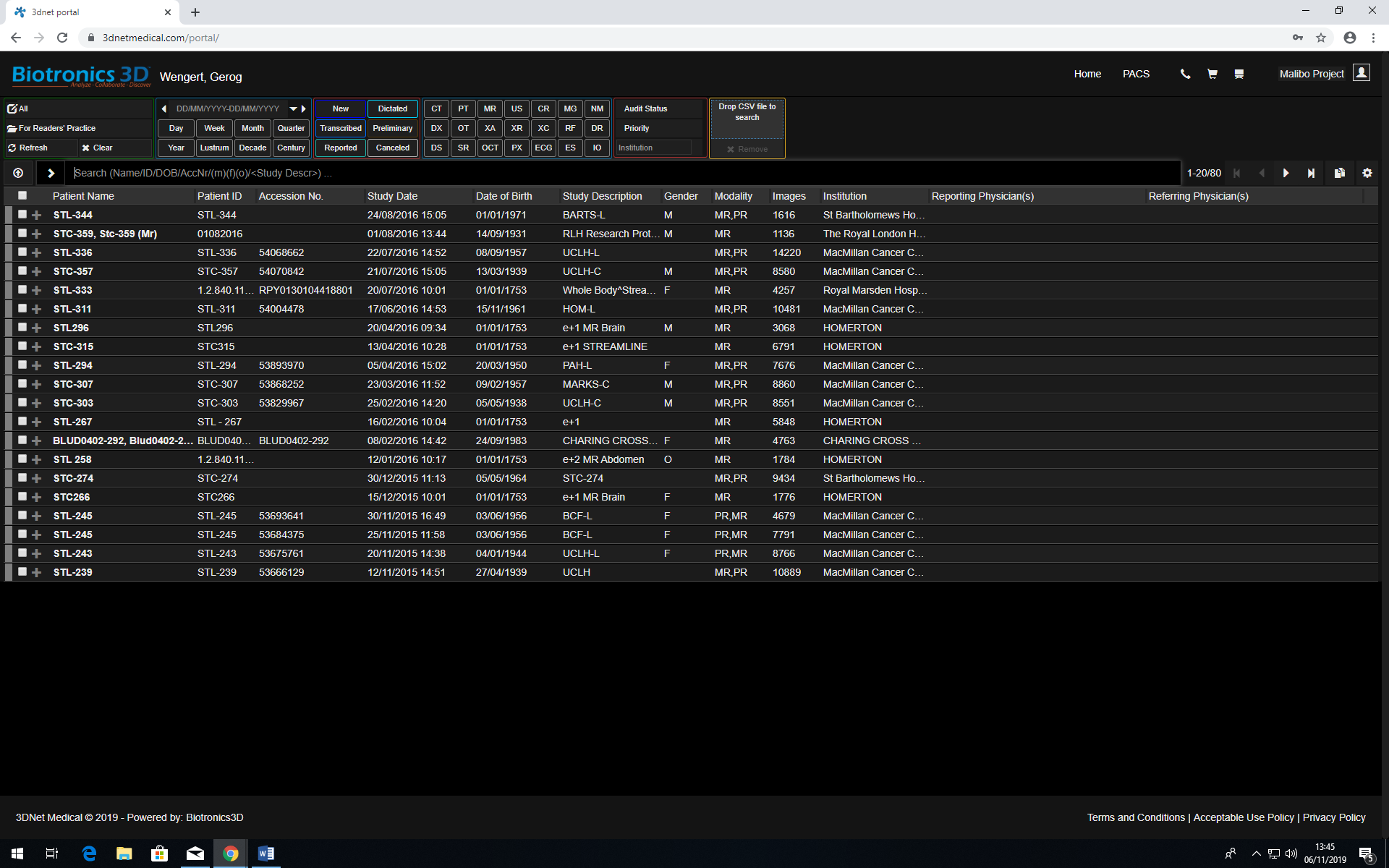


Chose the second icon from the left (**Series**) to open all given series


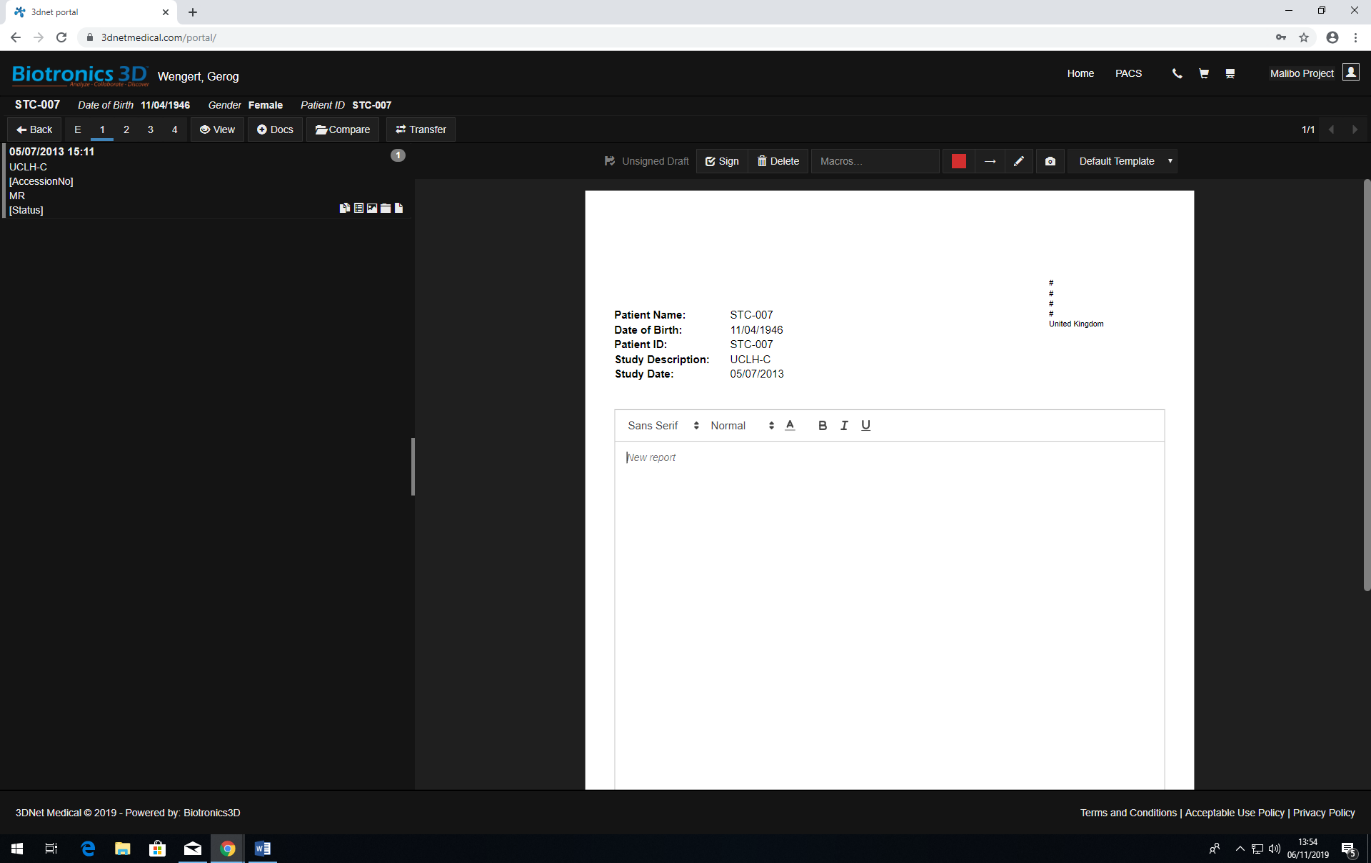


-----------------------------------------------------------------------------------------------------------------

Select the appropriate images stated on the CRF sheet (T2 ax stack, DW ax stack, ADC ax stack, ML output if available), as well as other appropriate series (T1 dynamic liver, lung, head/neck) by checking the icon box

Afterwards click **View** to open


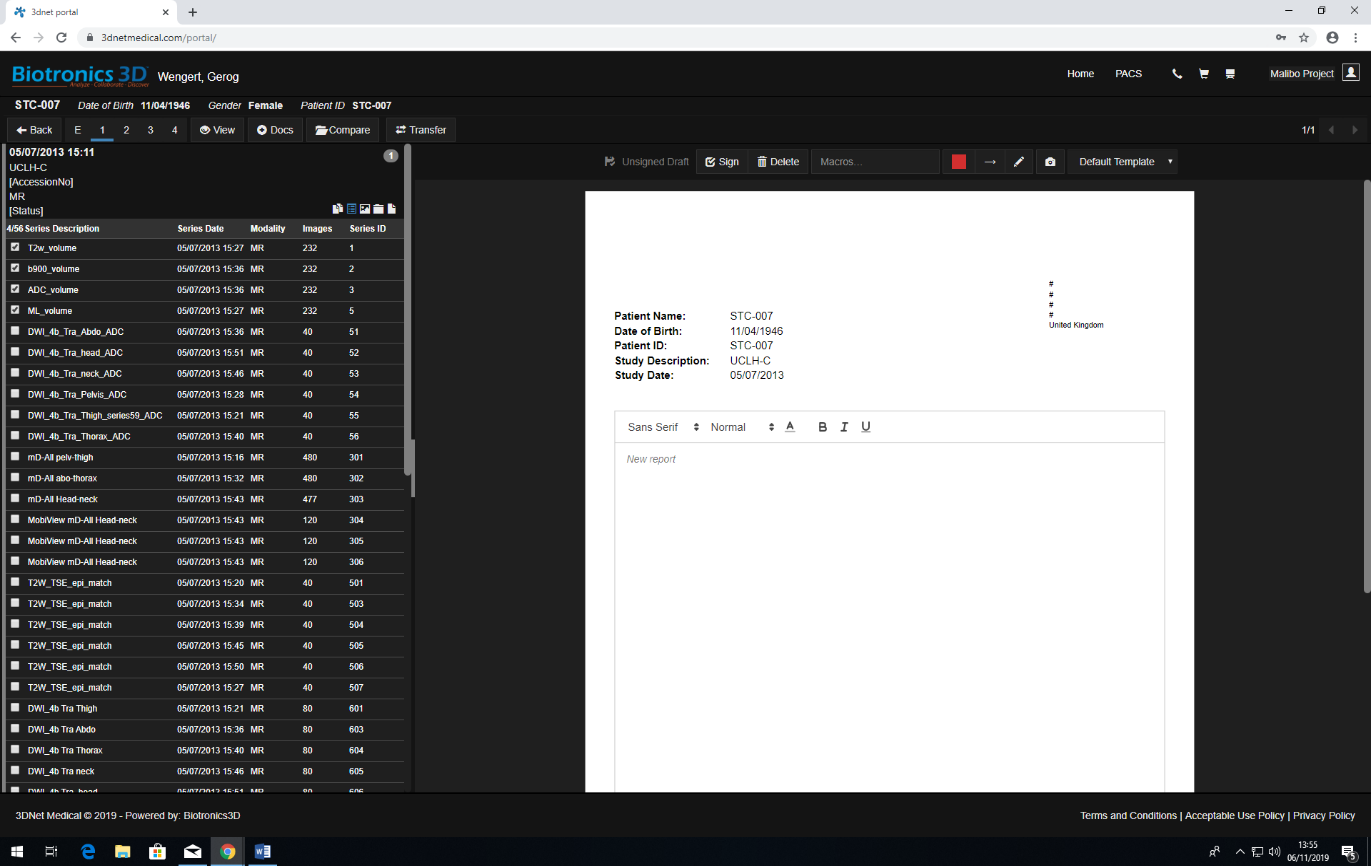


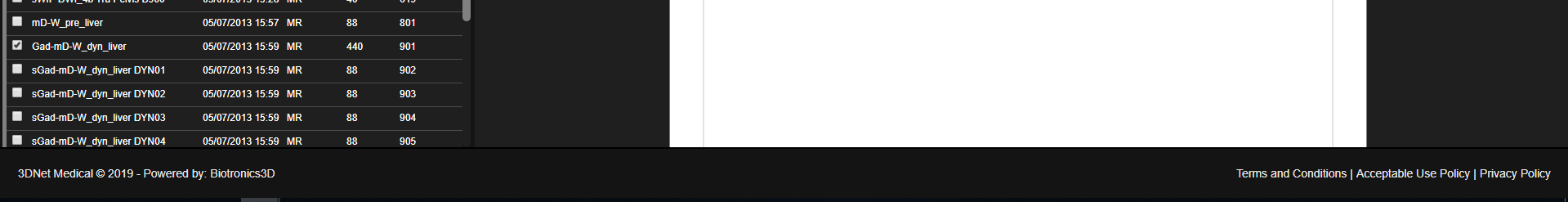


----------------------------------------------------------------------------------------------------------------

After a new window has opened, you can click **F11** for full screen mode


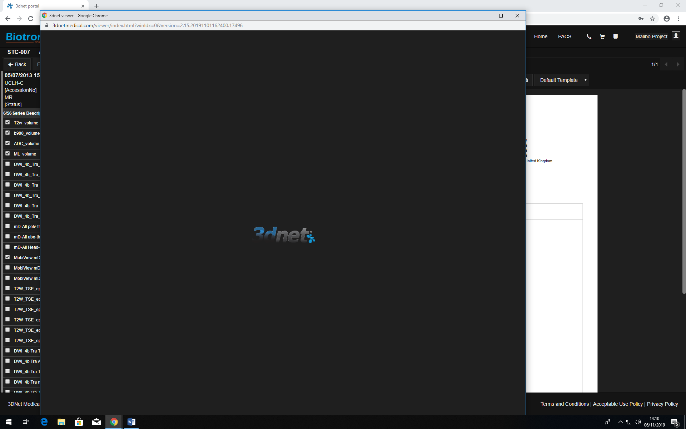

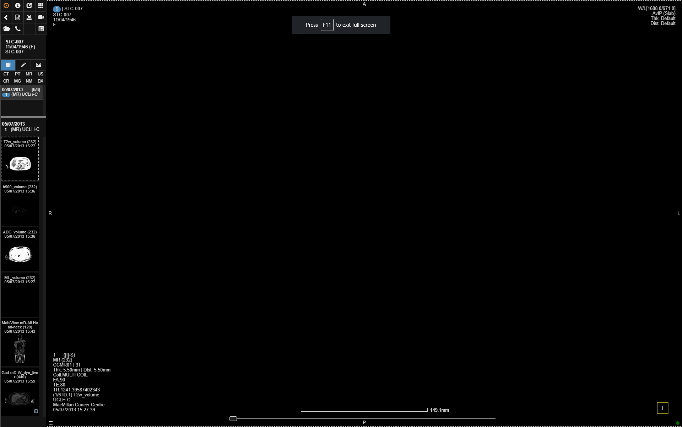


Use the indicated icon on the sidebar to open a dedicated PACS interface


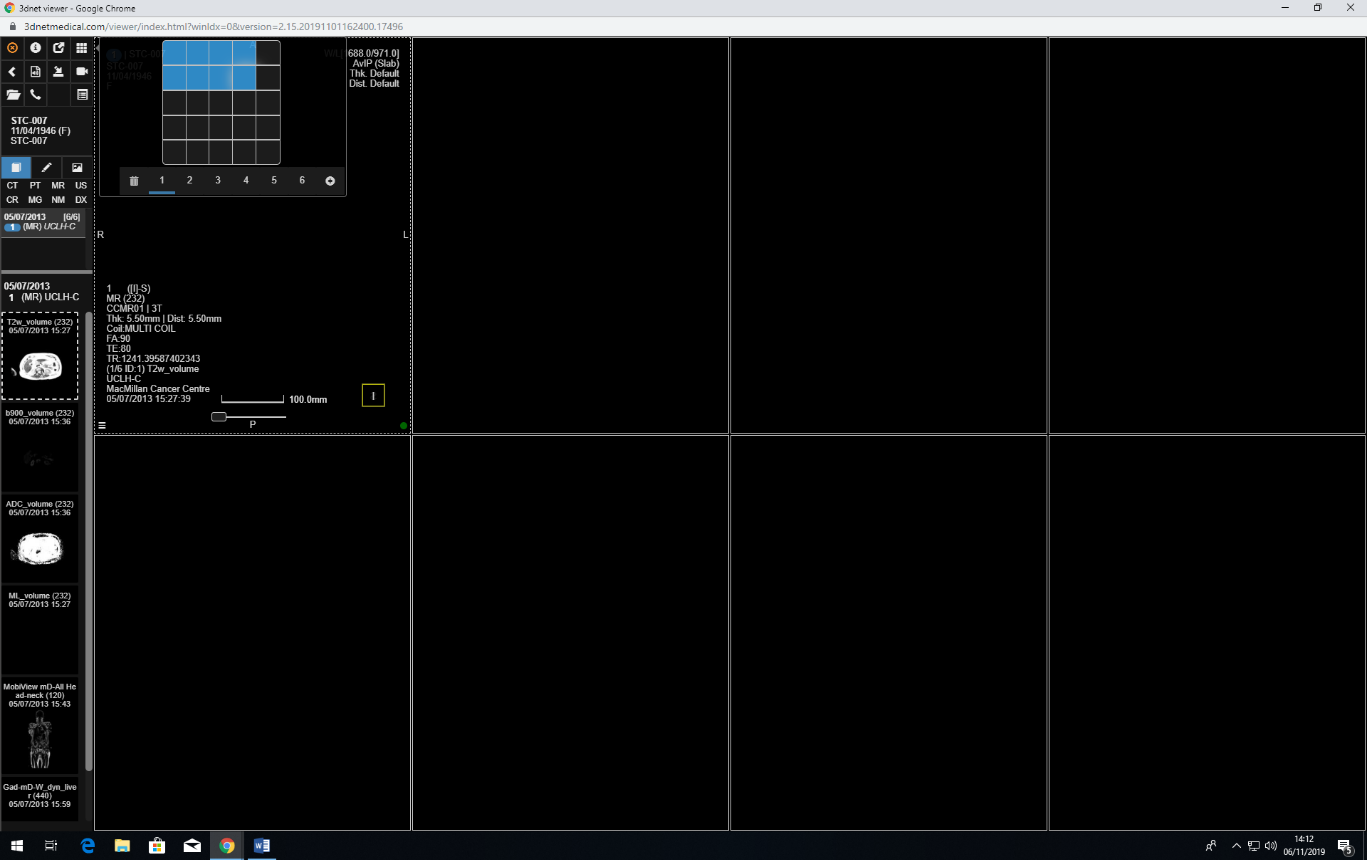


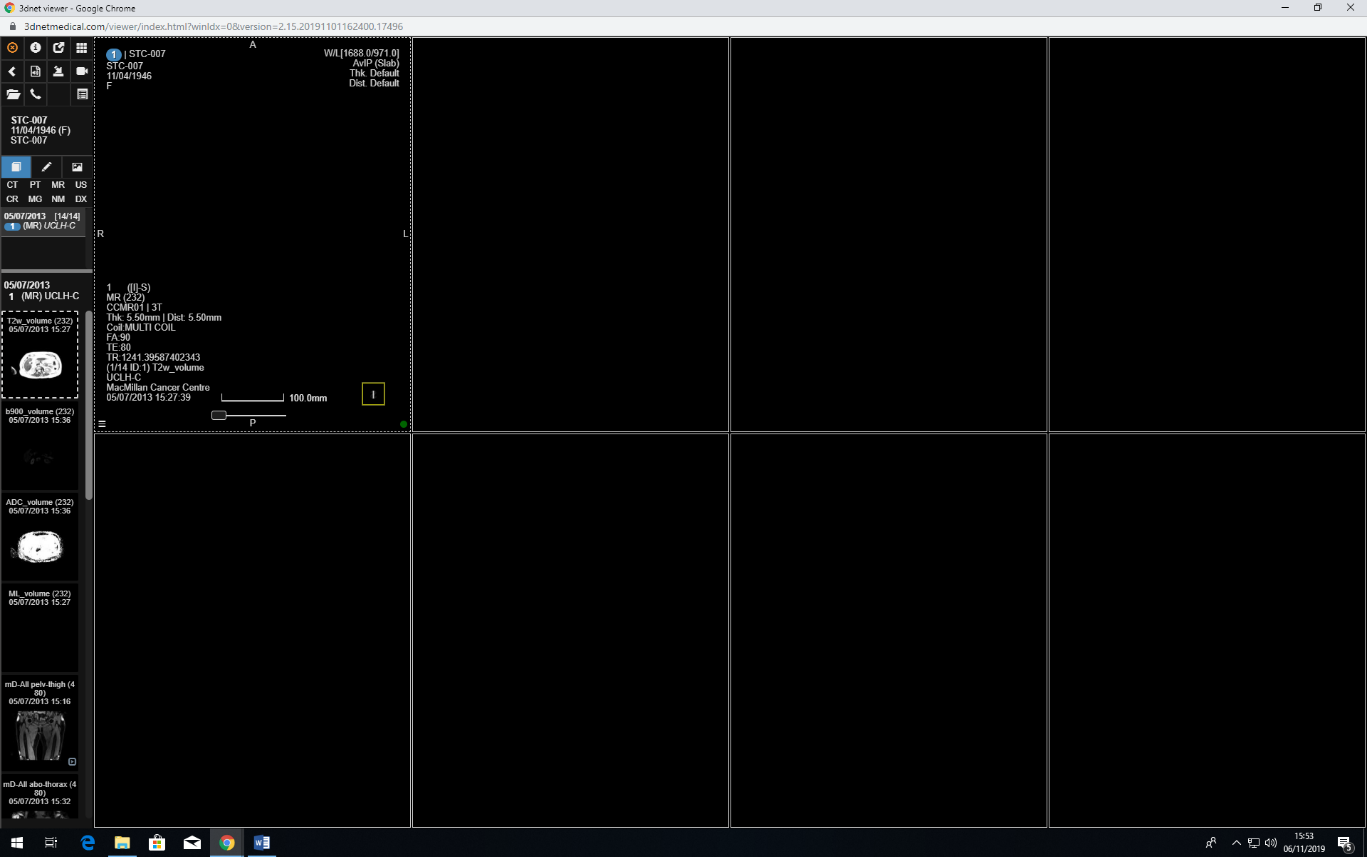


Choose sequences from the hanging protocol on the left sidebar by drag and drop.

The following image illustrates the recommended user layout for the readings

| T2 | DWI | ADC | T1 |
| --- | --- | --- | --- |
| ML | Liver | Lung | Brain |

**ML = Machine Learning algorithm, please use DM5 as your primary output,**

**RF5 is available as a secondary output for checking small lesions**

Once you have established your own preferred layout, you can connect all sequences to scroll them together via:

Position all images at the same anatomic level then you must keep **“Ctrl+Shift”** down and select each chosen sequence by clicking them. Each selected window will be marked with blue outlines (choose perhaps only the volumes/stacks and ML)

Choose **right mouse button** and select the icon on the open window to link them.

You have to hold **Ctrl+Shift** down until the link is complete!


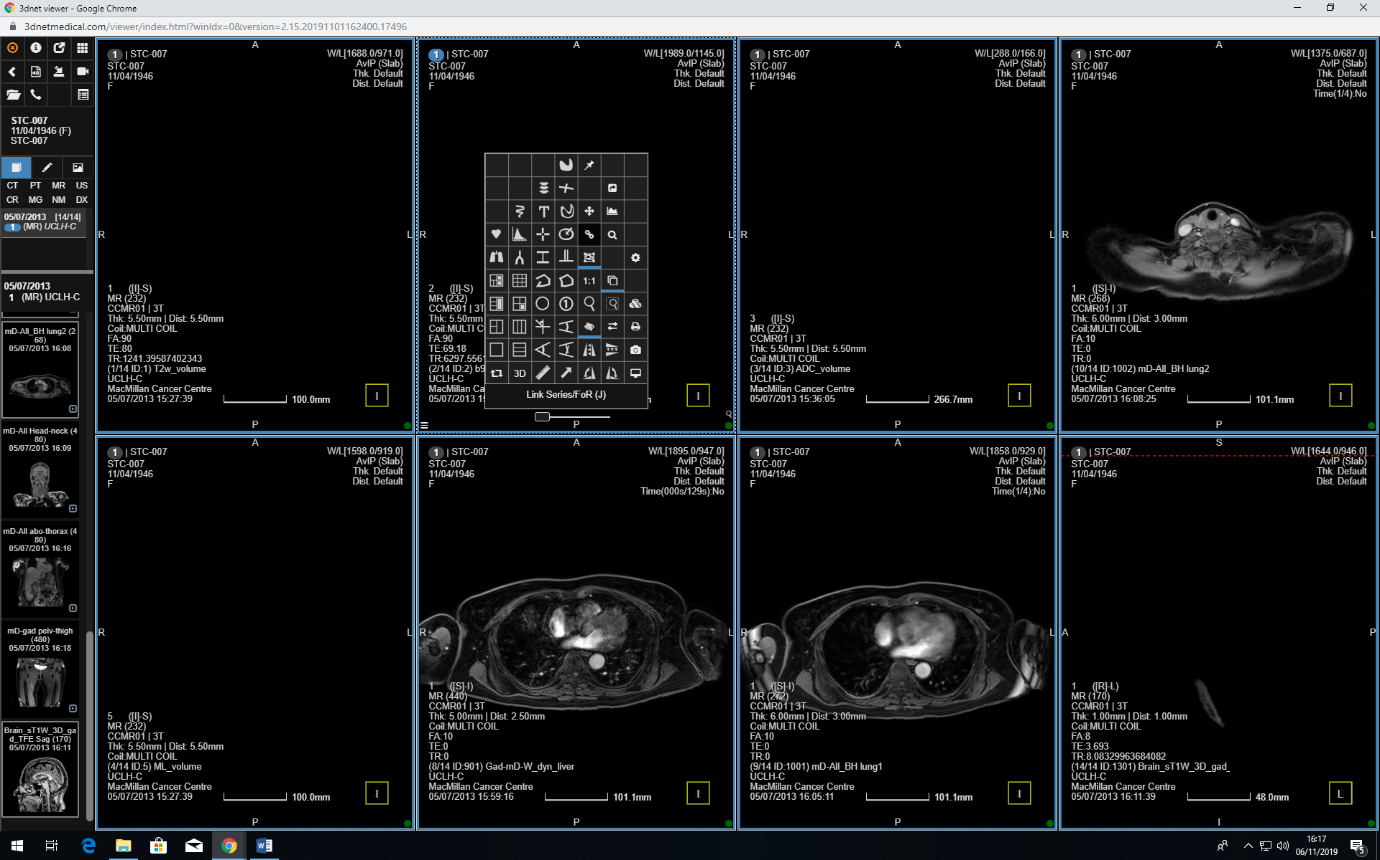


Now you can scroll through the images.

-----------------------------------------------------------------------------------------------------------------

For windowing, use **right mouse button** or select **W/L** in the right upper corner. This will open a new window, where the appropriate settings can be done.


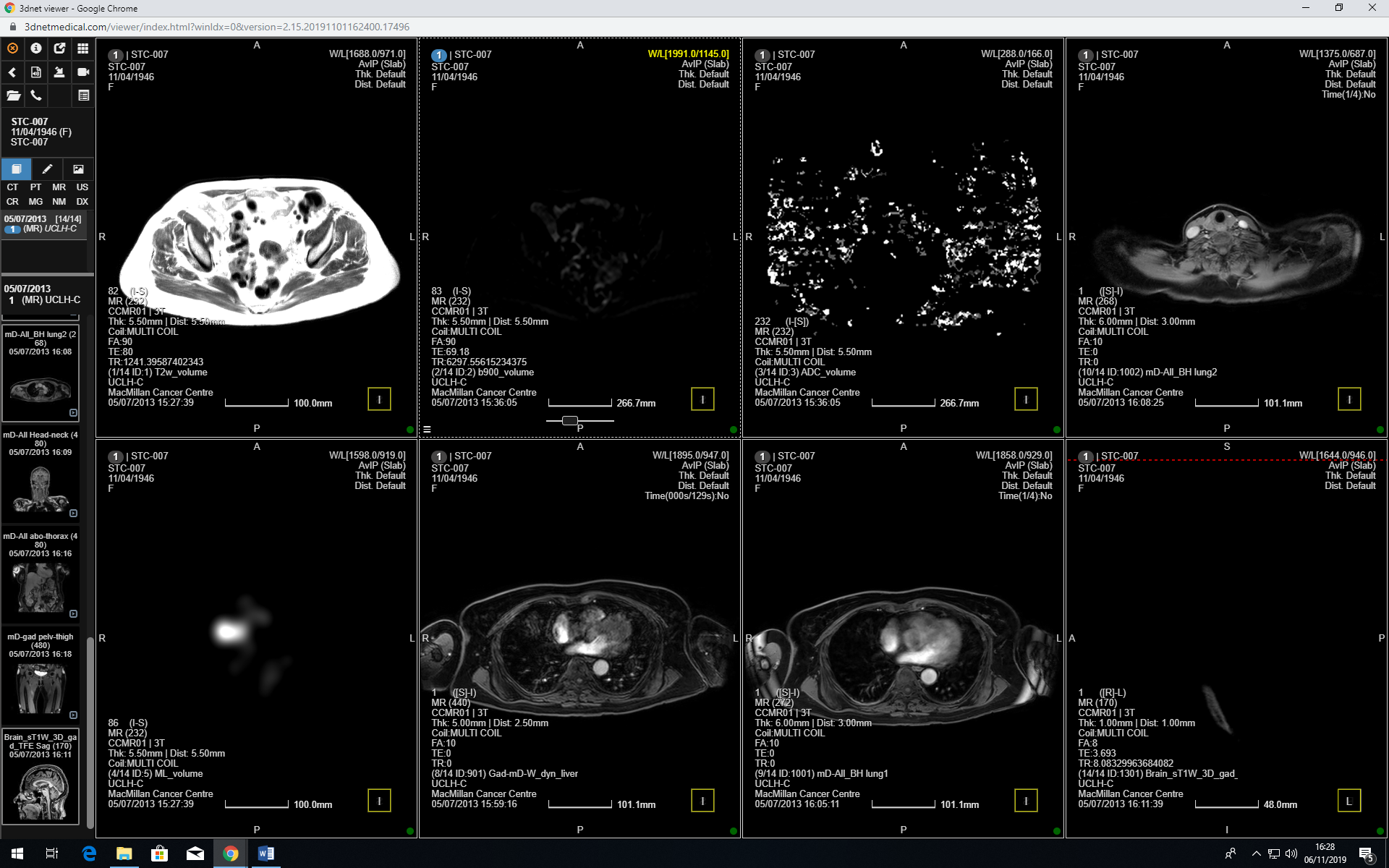

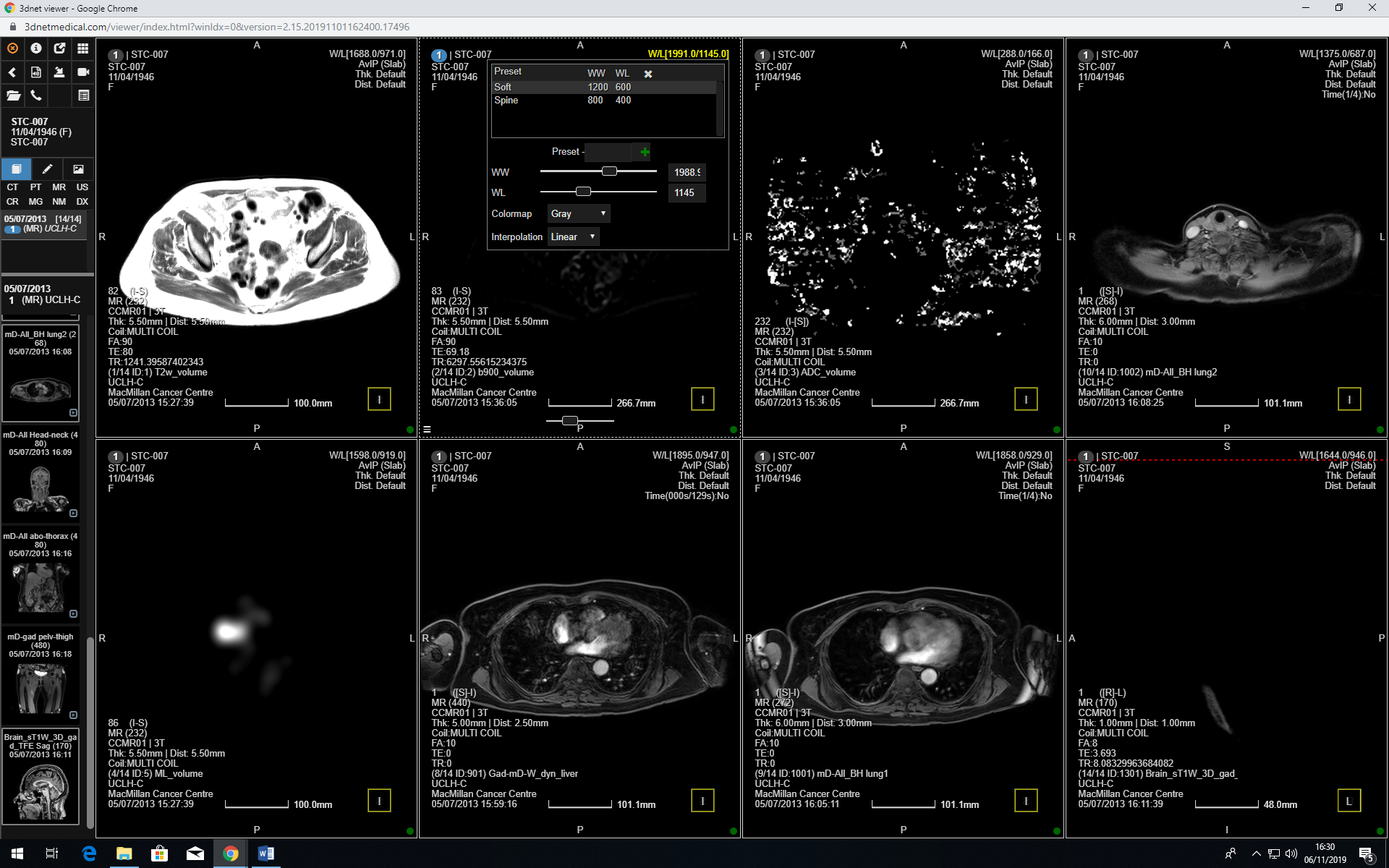

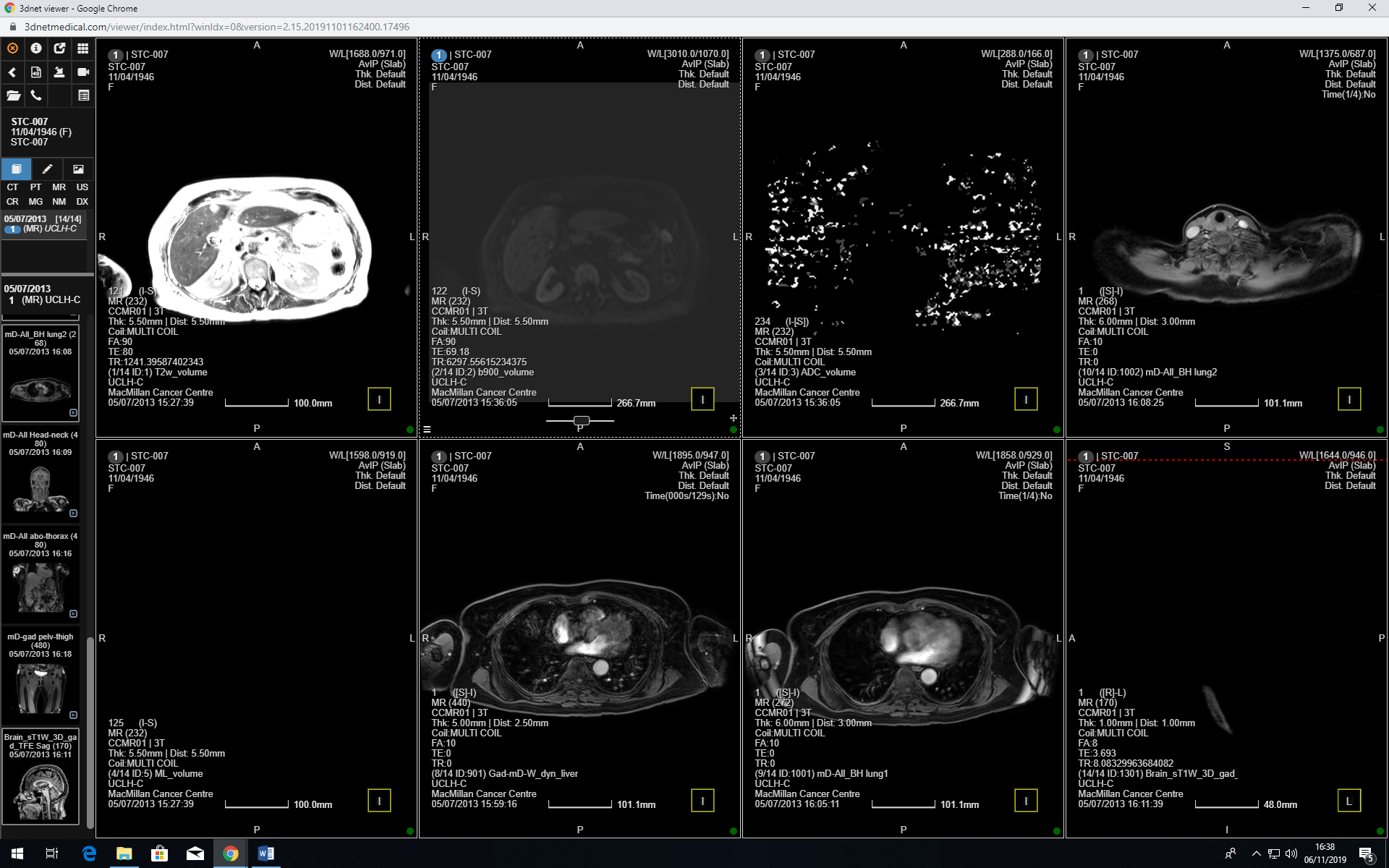


To merge the T2 and the ML sequences together keep again **Ctrl+Shift** down and select both sequences by clicking them.

Afterwards, click with the **right mouse button over the T2 image (!)** and select the icon as indicated.


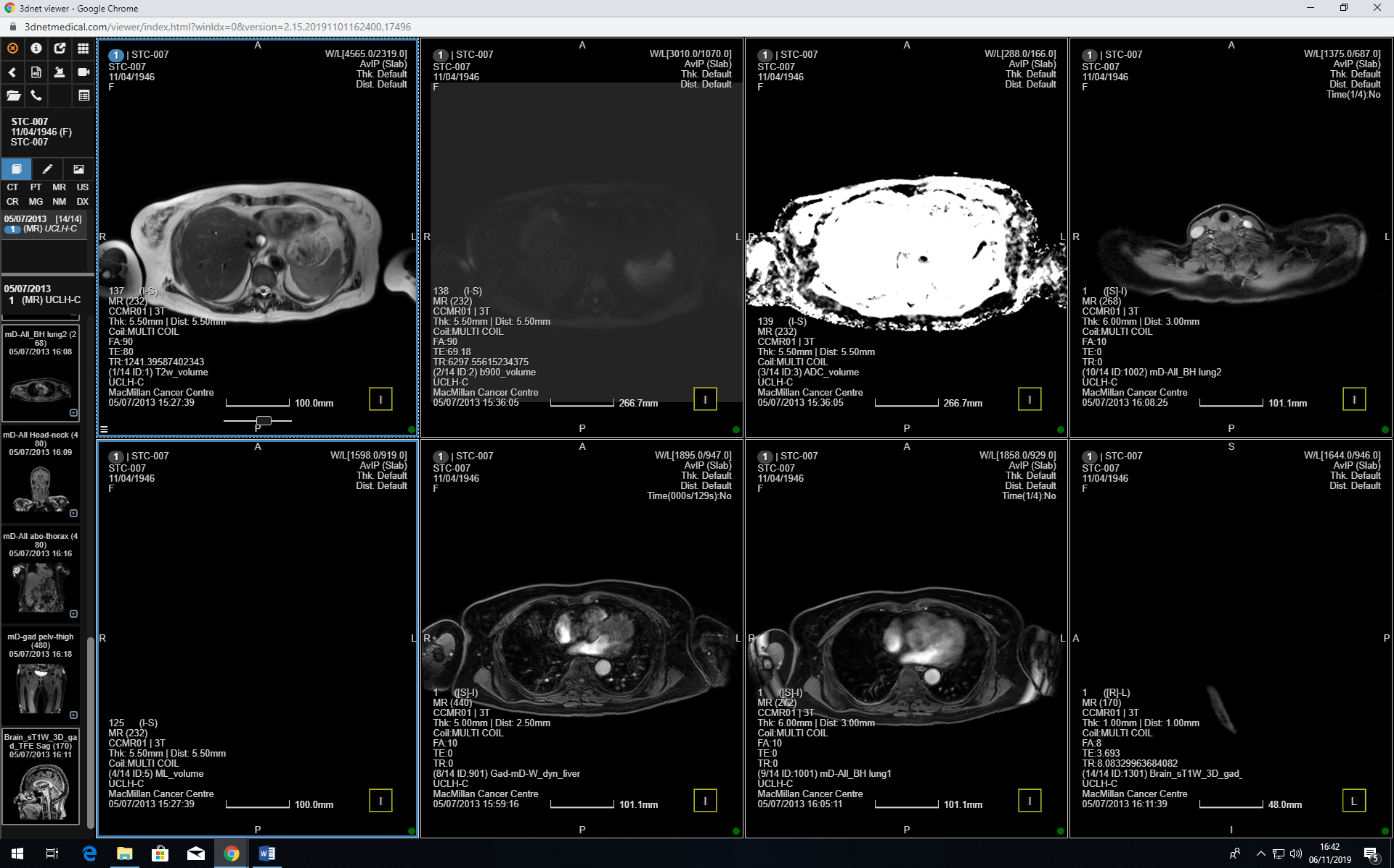

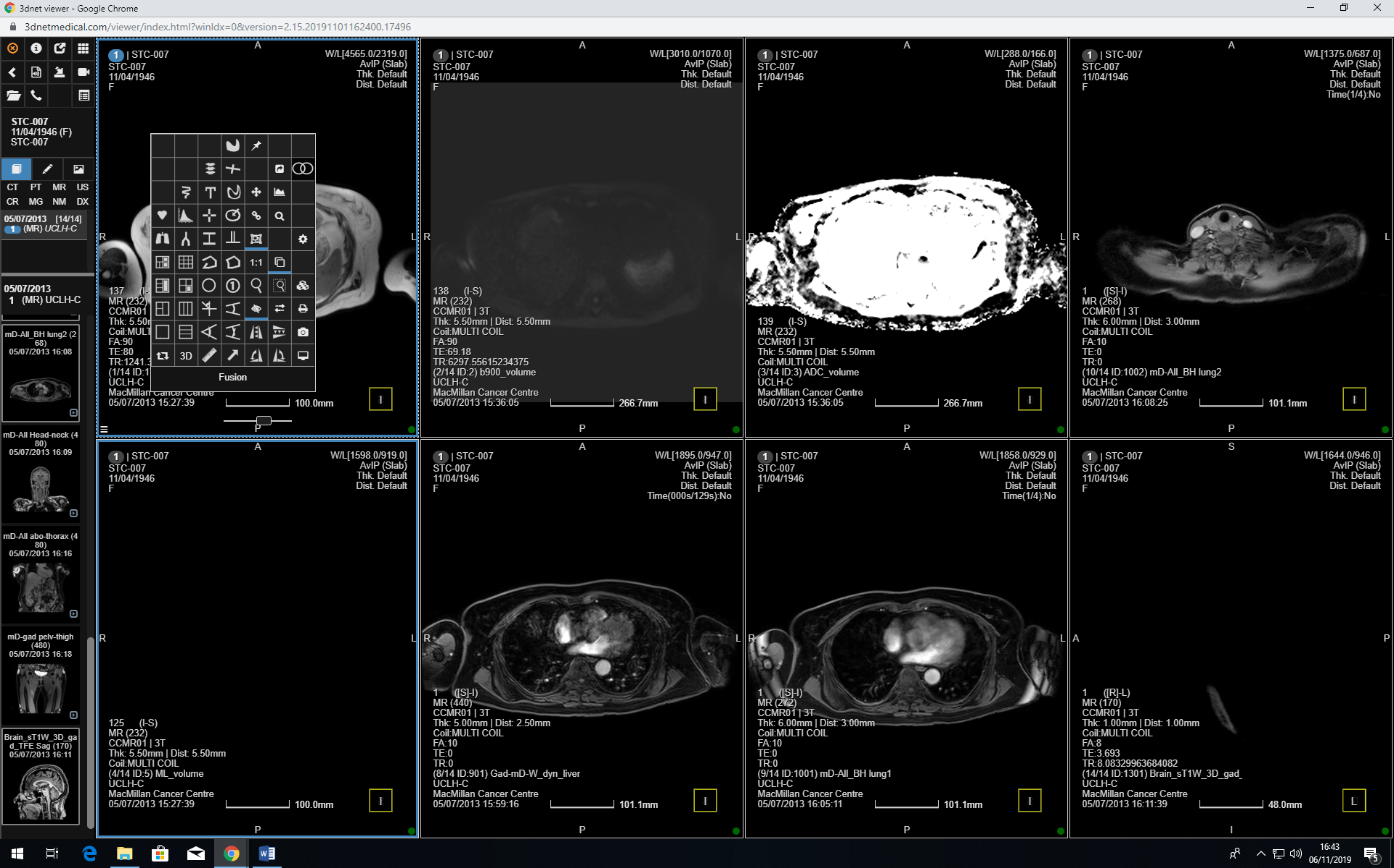

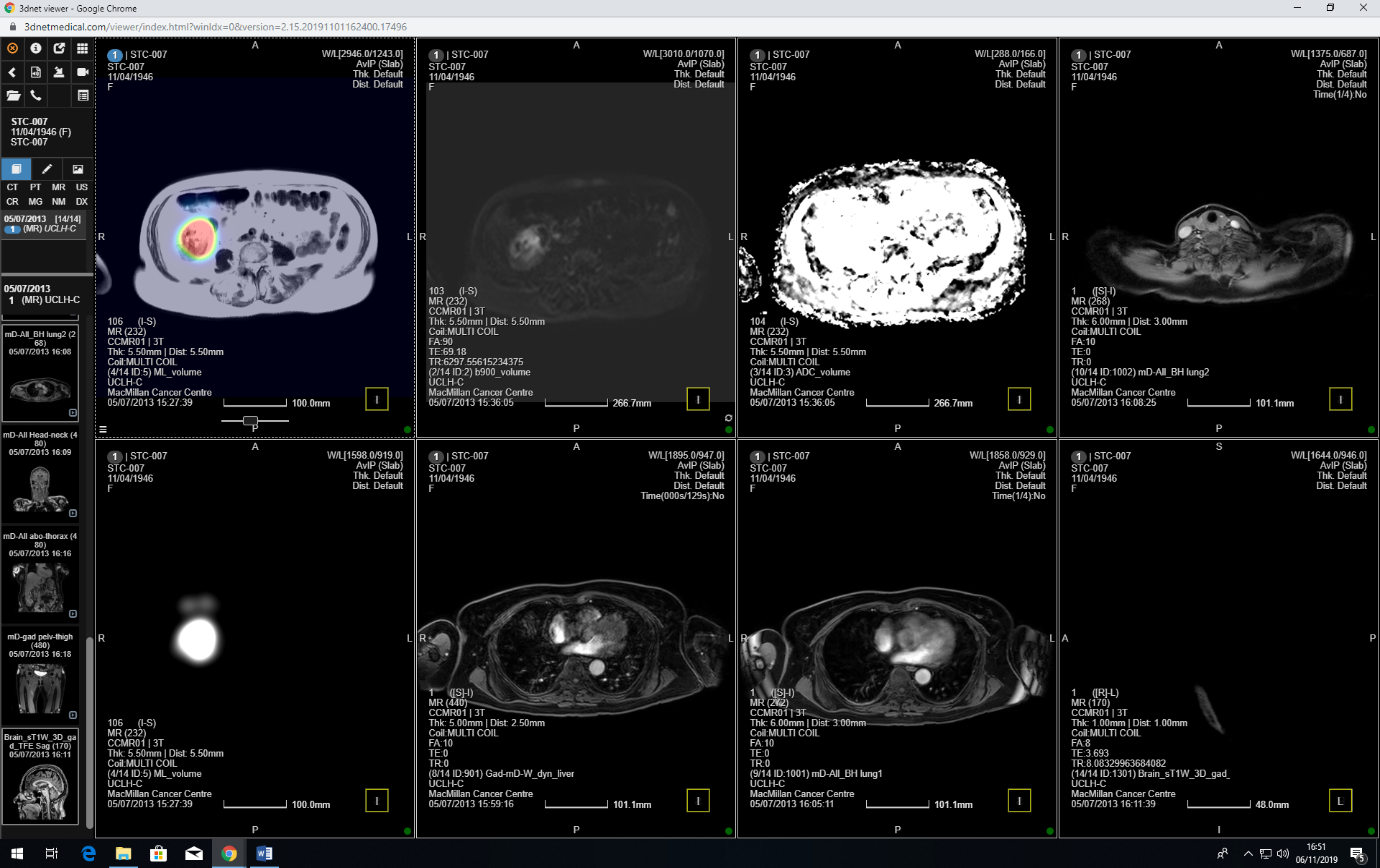


-----------------------------------------------------------------------------------------------------------------

Once you have created the merged image you can further adjust the balance to a certain side by selecting the icon again and adjust the threshold button on the right side. We recommend a 65% threshold selection.

*In case of improper windowing of the anatomic sequence (T2), as in this particular example*

*please load another T2 sequence into a different window*


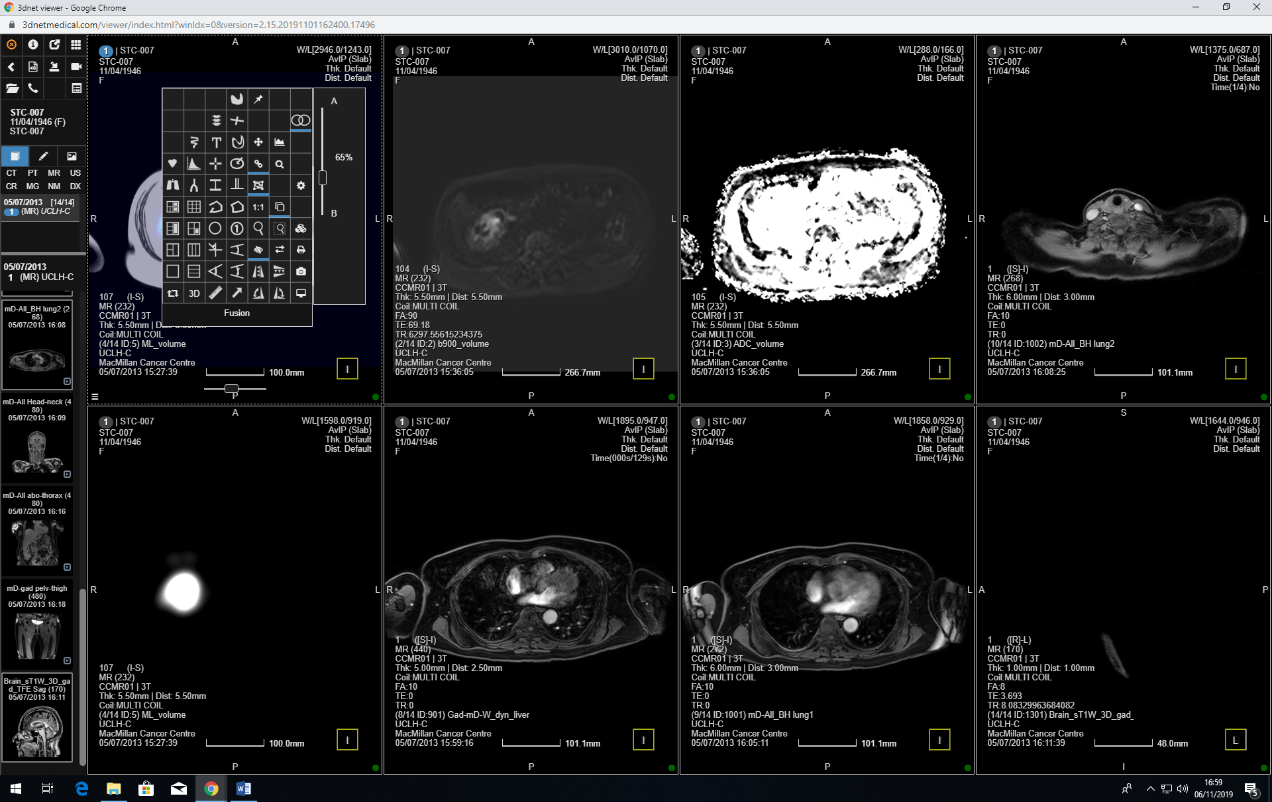

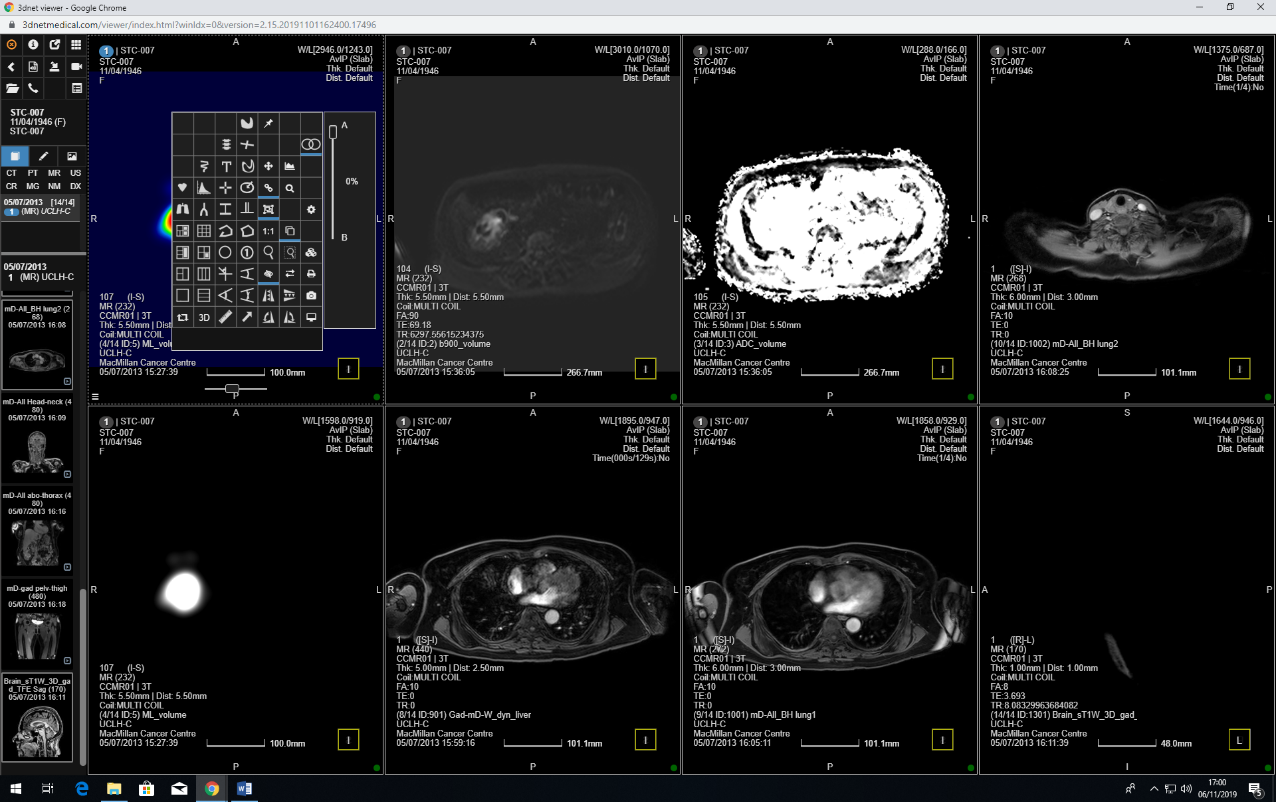

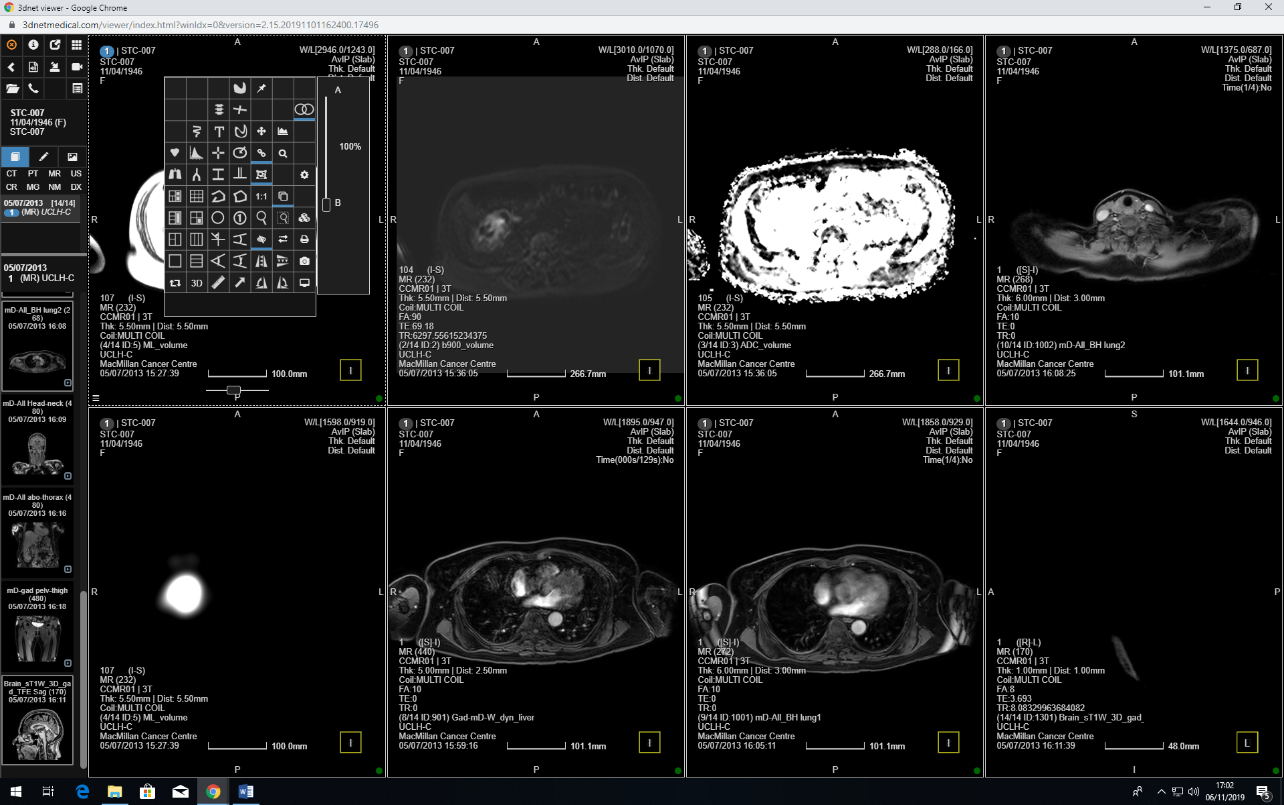


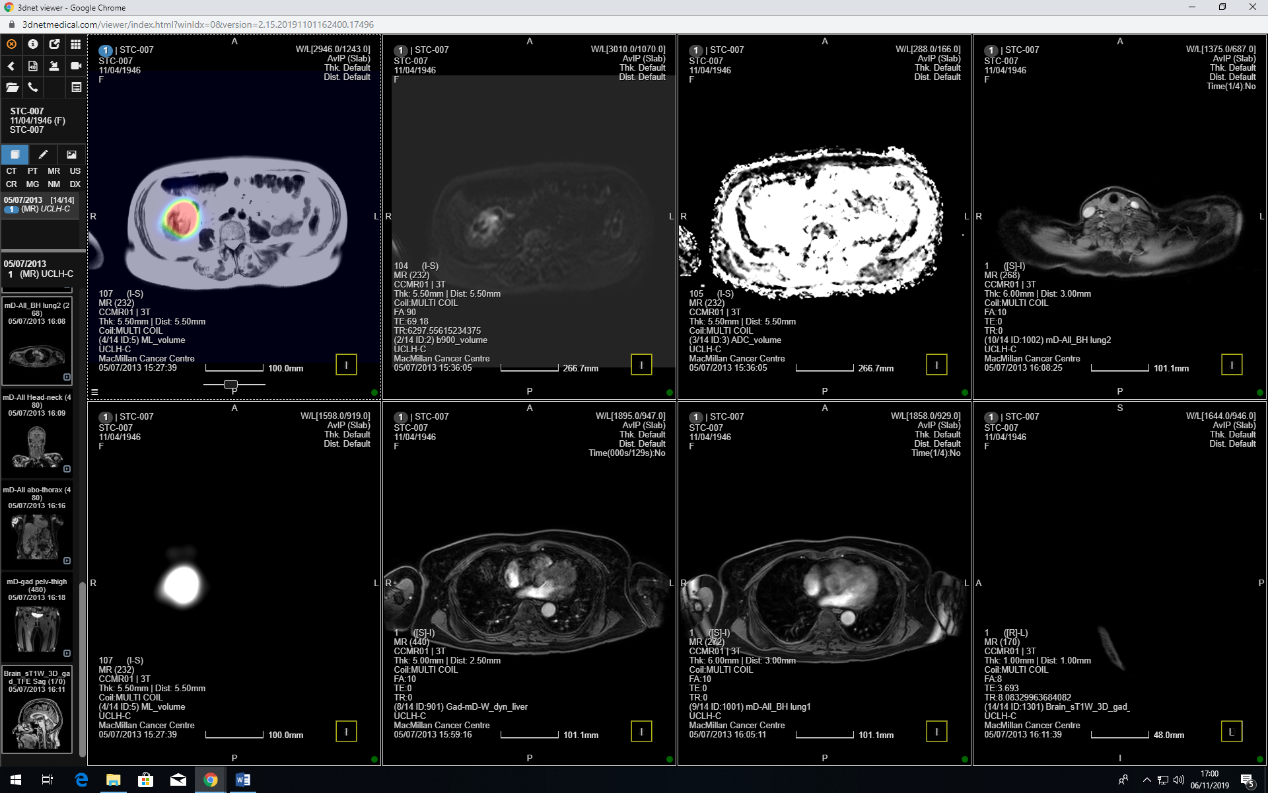

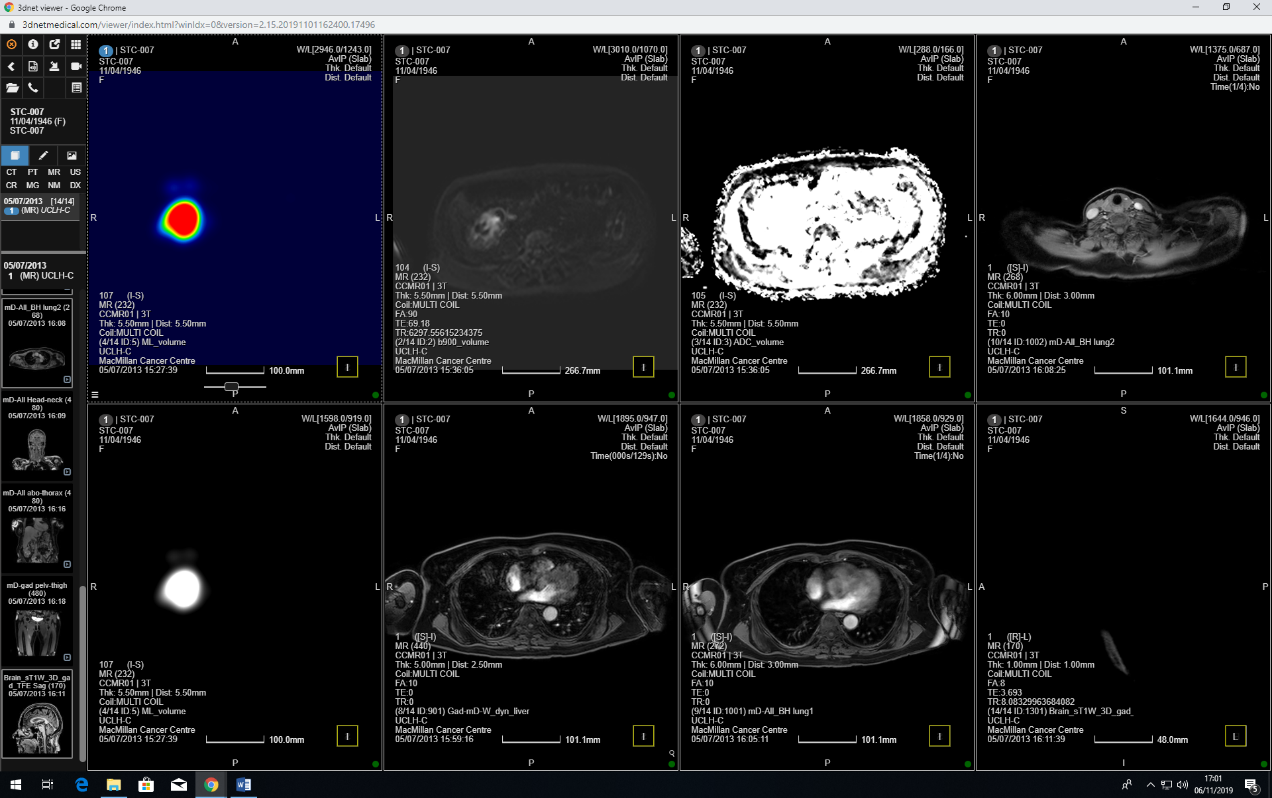

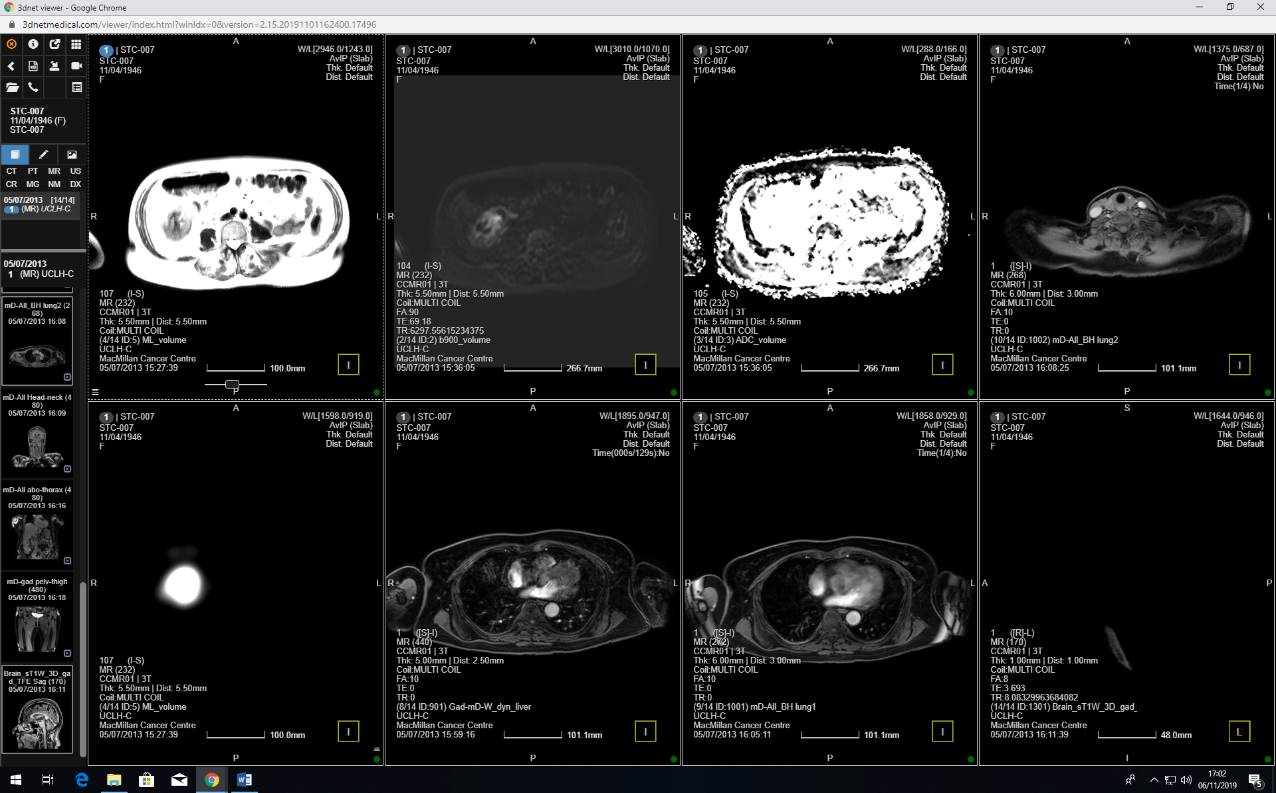


As all 80 training cases are from the Phase 2 trial have inconsistent sequences, there are some with four ML series! You can select and use all four in your training. Some cases have no ML output.

The DM5 should be merged together to the T2 sequence and used primarily for the reading as it has the highest specificity

The RF5 should be used secondarily and only to check for small lesions as it has high sensitivity, but low specificity.


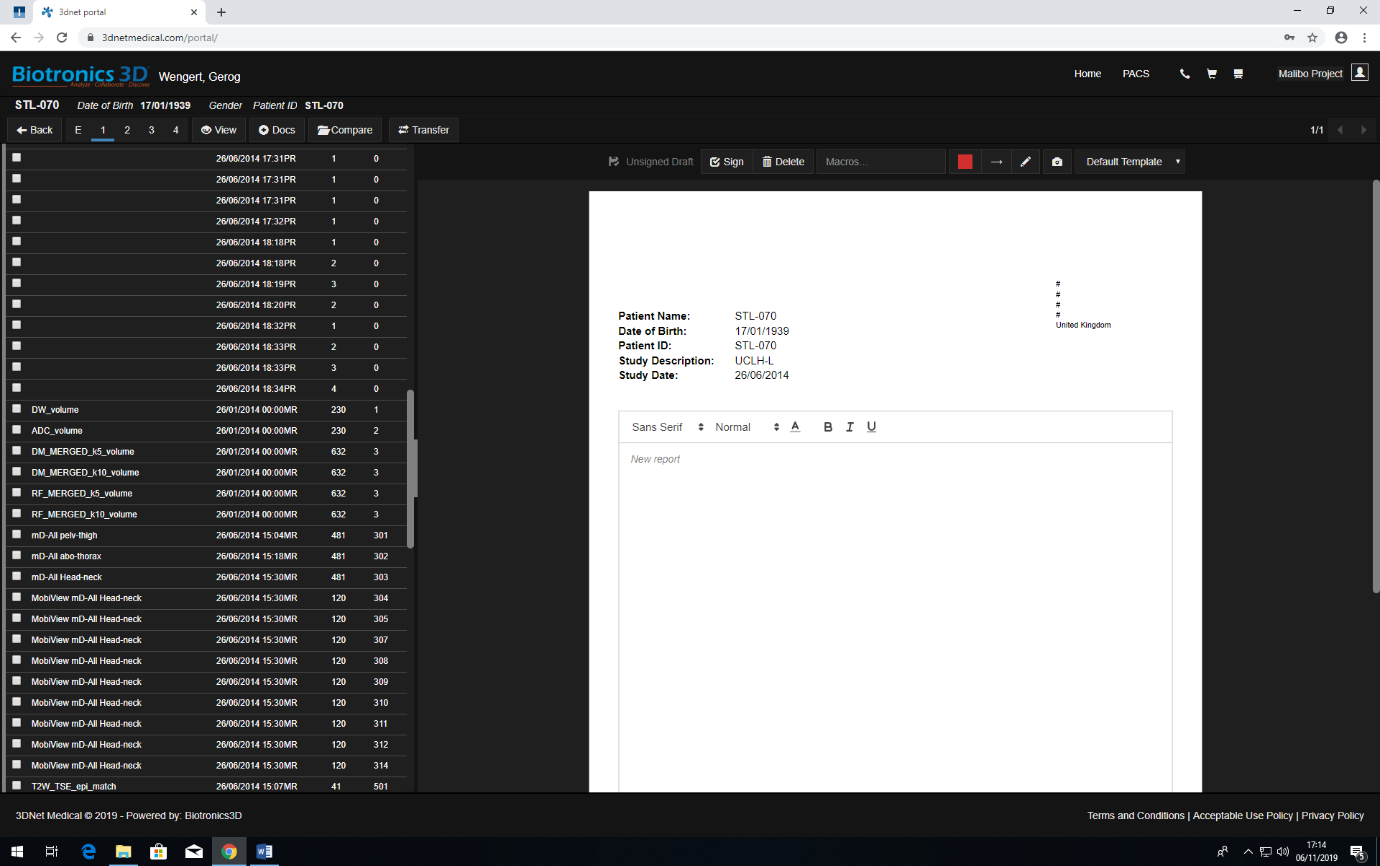


Scoring the ML output.

During your phase 3 read, you will be asked to give “your opinion” in relation to the primary tumour position and stage, nodal stage and presence and position of mets. Once “your opinion” is recorded, you will be asked to go back and record what the ML output was like – on a score of 1 (no colour at all – no probability of cancer lesion), 2 (blue green colour – low probability of cancer lesion), 3 (orange, moderate probability of cancer lesion), 4 (red, high probability of cancer lesion). See examples below, score 2 for bowel, score 3 for bowel and score 4 for posterior mediastinum.


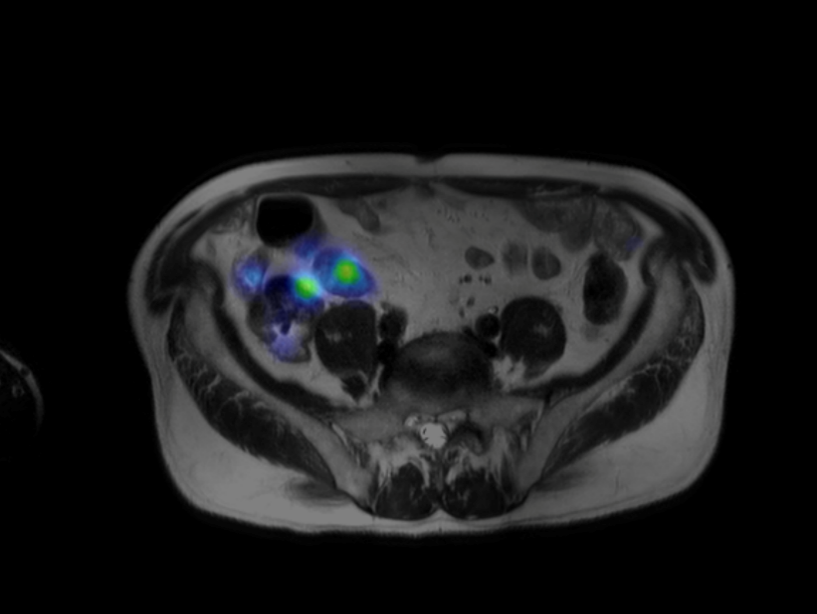


**
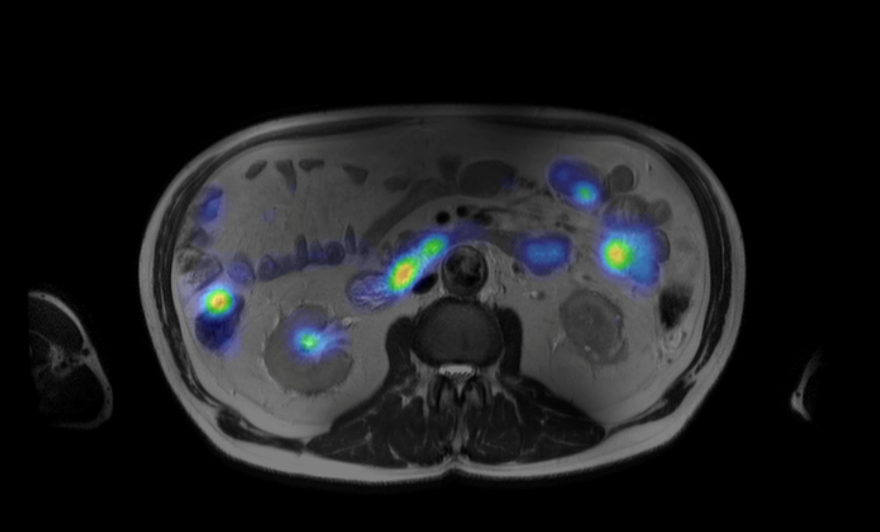
**

**
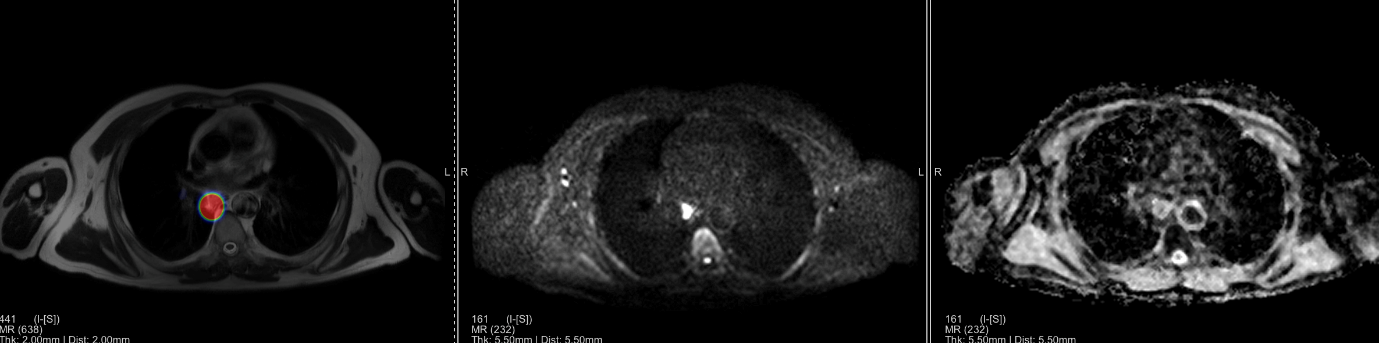
**
