# Supplementary material for: Development and Evaluation of Machine Learning in Whole-Body Magnetic Resonance Imaging for Detecting Metastases in Patients With Lung or Colon Cancer: A Diagnostic Test Accuracy Study
Source: Invest Radiol. 2023 Jun 26;58(12):823–31. doi: 10.1097/RLI.0000000000000996 (PMC10662596; doi:10.1097/RLI.0000000000000996)
Supplement: Supplementary file 3 [file ir-58-823-s003.docx]

**Supplemental Digital Content 4.**

Case report forms for reader study data collection

XXX STC CRF

DEVELOPMENT AND EVALUATION OF MACHINE LEARNING METHODS IN WHOLE BODY MR WITH DIFFUSION WEIGHTED IMAGING FOR STAGING OF PATIENTS WITH CANCER

**CASE REPORT FORM**

TRIAL NUMBER. XXXX-STC-____ ____ _____- ML _______(Y/N)

Please send ORIGINAL forms to:

[blinded]

General enquiries: Telephone: Email:

Co-ordinator:

Name of reader: _____________________________ Reader number: ­­­­_______

Date of read: _____________________________

Which reading round: round 1 /round 2 /round 3

Staging sheets can be provided to each reader at the time of the read.

IF ML OUTPUT IS AVAILABLE, THEN USE IT STRAIGHT AWAY AS WITH ANY AVAILABLE SEQUENCE. HOWEVER, IT IS ESSENTIAL THAT ML EVALUATION COLUMNS ARE NOT COMPLETED UNTIL THE CLINICAL READ IS FINISHED AND TIME OF READ IS RECORDED SO THAT WE CAN COMPARE WITH AND WITHOUT ML READING TIMES. THANK YOU.

Please sign to confirm that ML outputs will be completed after the clinical read:

Radiologist signature: _________________________

Exam read start time: _______:_________ (24 hour clock, hours, minutes)

**Images available? Please tick.**

|  | Available? | | Quality of sequence | | |
| --- | --- | --- | --- | --- | --- |
|  | Y | N | good | adequate | poor |
| T2 axial stack |  |  |  |  |  |
| DW axial stack |  |  |  |  |  |
| ADC axial stack |  |  |  |  |  |
| ML output available |  |  | --------------- | -------------- | --------------- |
|  |  |  |  |  |  |
| T1 axial stack |  |  |  |  |  |
| T1 coronal |  |  |  |  |  |
| Liver with contrast |  |  |  |  |  |
| Brain with contrast |  |  |  |  |  |
| T1FS post contrast (body) |  |  |  |  |  |

Comment:

**PRIMARY TUMOUR DETECTION**

Based on all available information, including ML output if available

Note: if a tumour crosses an anatomical boundary, please choose a single site of tumour (you can add a comment if you wish). If there are two separate primary tumours, you can add this as a second site.

|  | **1**  **No primary tumour identified at this site** | **2**  **Probably no primary tumour at this site** | **3**  **Probably primary tumour at this site** | **4**  **Highly likely primary tumour at this site** | **DM5 score**  **1-4**  **(NA if no ML output)** | RF5 used?  Please tick at any site where this was used for detection |
| --- | --- | --- | --- | --- | --- | --- |
| **Rectum** |  |  |  |  |  |  |
| **Sigmoid** |  |  |  |  |  |  |
| **Descending colon** |  |  |  |  |  |  |
| **Transverse colon** |  |  |  |  |  |  |
| **Ascending colon** |  |  |  |  |  |  |
| **Caecum** |  |  |  |  |  |  |
| **Max dimension (mm) if measurable primary tumour (NA if no primary tumour seen)** |  |  |  |  |  |  |

**Comment:** (optional if second tumour or other comment):

**T stage (as per TNM version used in STREAMLINE study) based on all available information.** If you identify a primary tumour at any site, with any confidence level, please tick one cell only; if no primary tumour identified at any site, indicate this in appropriate cell. If two primary lesions are identified, then please stage according to the highest stage. Add comment if you wish.

**Note:** if uncertain of stage then please select the most likely stage with corresponding level of confidence/uncertainty.

| *tick 1 box in this table | **1**  **very low confidence** | **2**  **low confidence** | **3**  **reasonable confidence** | **4**  **high confidence** |
| --- | --- | --- | --- | --- |
| **No primary tumour identified** |  |  |  |  |
| **T1** |  |  |  |  |
| **T2** |  |  |  |  |
| **T3** |  |  |  |  |
| **T4** |  |  |  |  |

**Comment:** (optional)

**REGIONAL NODES:**

Presence of nodal metastases based on all available information, including ML output if available

**Nodal stage (as per TNM version used in STREAMLINE study):**

Note: if uncertain of stage then please select the most likely stage with corresponding level of confidence/uncertainty

| * tick 1 box in this table | **1**  **very low confidence** | **2**  **low confidence** | **3**  **reasonable confidence** | **4**  **high confidence** | **DM5 stage**  **(1-4)** | RF5 used?  Please tick at any site where this was used for detection |
| --- | --- | --- | --- | --- | --- | --- |
| **N0** |  |  |  |  |  |  |
| **N1** |  |  |  |  |  |  |
| **N2** |  |  |  |  |  |  |

**Comment:** (optional)

**METASTASES: NON-SKELETAL SITES**

Based on all available information, including ML output if available. If “Negative 1 or 2” is selected by reader, no measurement required, even if ML score >2. If “Positive 3 or 4” is selected by reader, then size(s) should be given.

| Presence or absence of metastasis based on all available information  *-Please tick* | Negative  1- definitely not present  2- probably not present | | Positive  3- probably present  4- highly likely present | | Size of largest organ deposit *(mm)* | | Size of second largest organ deposit *(mm)*  *(if not applicable put N/A)* | | Number of additional deposits  ≥6mm  *(if ≤10, state number. If >10 state, >10)*  *(if not applicable put N/A)* | | Number of additional deposits  <6mm  *(if ≤10, state number. If >10 state, >10)*  *(if not applicable put N/A)* | | RF5 used?  Please tick at any site where this was used for detection |
| --- | --- | --- | --- | --- | --- | --- | --- | --- | --- | --- | --- | --- | --- |
|  | **Negative** | | **Positive** | |  |  |  |  |  |  |  |  |  |
|  | 1 | 2 | 3 | 4 |  |  |  |  |  |  |  |  |  |
|  |  |  |  |  |  | DM5 score 1-4 |  | DM5 score  1-4 |  | DM5  score  1-4 |  | DM5 score  1-4 |  |
| Brain |  |  |  |  |  |  |  |  |  |  |  |  |  |
| Lung (L) |  |  |  |  |  |  |  |  |  |  |  |  |  |
| Lung (R) |  |  |  |  |  |  |  |  |  |  |  |  |  |
| Pleura (L) |  |  |  |  |  |  |  |  |  |  |  |  |  |
| Pleura (R) |  |  |  |  |  |  |  |  |  |  |  |  |  |
| Liver (left lobe) |  |  |  |  |  |  |  |  |  |  |  |  |  |
| Liver (right lobe) |  |  |  |  |  |  |  |  |  |  |  |  |  |
| Spleen |  |  |  |  |  |  |  |  |  |  |  |  |  |
| Adrenal (L) |  |  |  |  |  |  |  |  |  |  |  |  |  |
| Adrenal (R) |  |  |  |  |  |  |  |  |  |  |  |  |  |
| Kidney (L) |  |  |  |  |  |  |  |  |  |  |  |  |  |
| Kidney (R) |  |  |  |  |  |  |  |  |  |  |  |  |  |
| Pancreas |  |  |  |  |  |  |  |  |  |  |  |  |  |
| Mesentery/peritoneum |  |  |  |  |  |  |  |  |  |  |  |  |  |
| Bowel |  |  |  |  |  |  |  |  |  |  |  |  |  |
| Soft tissue neck/chest |  |  |  |  |  |  |  |  |  |  |  |  |  |
| Soft tissue abdomen/pelvis |  |  |  |  |  |  |  |  |  |  |  |  |  |
| Nodal *(Non regional - Please state site; NA if no other nodal site)*________________ |  |  |  |  |  |  |  |  |  |  |  |  |  |
| Other *(Please state/ or NA if no other site)*________________ |  |  |  |  |  |  |  |  |  |  |  |  |  |

**Comment:** (optional)

**METASTASES: SKELETAL SITES**

Based on all available information, including ML output if available. If “Negative 1 or 2” is selected by reader, no measurement required, even if ML score >2. If “Positive 3 or 4” is selected by reader, then size(s) should be given.

| Presence or absence of metastasis based on all available information  *-Please tick* | Negative  1- definitely not present  2- probably not present | | Positive  3- probably present  4- highly likely present | | Size of largest organ deposit *(mm)* | | Size of second largest organ deposit *(mm)*  *(if not applicable put N/A)* | | Number of additional deposits  ≥6mm  *(if ≤10, state number. If >10 state, >10)*  *(if not applicable put N/A)* | | Number of additional deposits  <6mm  *(if ≤10, state number. If >10 state, >10)*  *(if not applicable put N/A)* | | RF5 used?  Please tick at any site where this was used for detection |
| --- | --- | --- | --- | --- | --- | --- | --- | --- | --- | --- | --- | --- | --- |
|  | **Negative** | | **Positive** | |  |  |  |  |  |  |  |  |  |
|  | 1 | 2 | 3 | 4 |  |  |  |  |  |  |  |  |  |
|  |  |  |  |  |  | DM5 score  1-4 |  | DM5 score  1-4 |  | DM5 score  1-4 |  | DM5 score  1-4 |  |
| Skull |  |  |  |  |  |  |  |  |  |  |  |  |  |
| Cervical spine |  |  |  |  |  |  |  |  |  |  |  |  |  |
| Thoracic spine |  |  |  |  |  |  |  |  |  |  |  |  |  |
| Lumbar spine |  |  |  |  |  |  |  |  |  |  |  |  |  |
| Pelvis |  |  |  |  |  |  |  |  |  |  |  |  |  |
| Sternum |  |  |  |  |  |  |  |  |  |  |  |  |  |
| Clavicle/Scapula (L) |  |  |  |  |  |  |  |  |  |  |  |  |  |
| Clavicle/Scapula (R) |  |  |  |  |  |  |  |  |  |  |  |  |  |
| Ribs (L) |  |  |  |  |  |  |  |  |  |  |  |  |  |
| Ribs (R) |  |  |  |  |  |  |  |  |  |  |  |  |  |
| Other *(Please State or NA)*    _________________ |  |  |  |  |  |  |  |  |  |  |  |  |  |

**Additional comments** (e.g. 2^nd^ primary, incidental benign findings) (optional)

Exam read end time: _________:_______ (24-hour clock, hours, minutes)

Now go back and fill in ML scores. Please tick.

Now go back to check table on page 2 is completed. Please tick.

CRF completed by (scribe): Signature:

CRF completed by (reader): Signature:

Date:

# XXX STL CRF

**XXX Study**

DEVELOPMENT AND EVALUATION OF MACHINE LEARNING METHODS IN WHOLE BODY MR WITH DIFFUSION WEIGHTED IMAGING FOR STAGING OF PATIENTS WITH CANCER

**CASE REPORT FORM**

TRIAL NUMBER. XXX-STL-____ ____ _____- ML _______(Y/N)

Please send ORIGINAL forms to:

[blinded]

General enquiries: Telephone: Email:

Co-ordinator:

Name of reader: _____________________________ Reader number:_______

Date of read: _____________________________

Which reading round: round 1 /round 2 /round 3

Staging sheets can be provided to each reader at the time of the read.

IF ML OUTPUT IS AVAILABLE, THEN USE IT STRAIGHT AWAY AS WITH ANY AVAILABLE SEQUENCE. HOWEVER, IT IS ESSENTIAL THAT ML EVALUATION COLUMNS ARE NOT COMPLETED UNTIL THE CLINICAL READ IS FINISHED AND TIME OF READ IS RECORDED SO THAT WE CAN COMPARE WITH AND WITHOUT ML READING TIMES. THANK YOU.

Please sign to confirm that ML outputs/scores will be completed after the clinical read:

Radiologist signature: _________________________

Exam read start time: _______:_________ (24 hour clock, hours, minutes)

**Images available? Please tick.**

|  | Available? | | Quality of sequence | | |
| --- | --- | --- | --- | --- | --- |
|  | Y | N | good | adequate | poor |
| T2 axial stack |  |  |  |  |  |
| DW axial stack |  |  |  |  |  |
| ADC axial stack |  |  |  |  |  |
| ML output available |  |  | --------------- | -------------- | -------------- |
|  |  |  |  |  |  |
| T1 axial stack |  |  |  |  |  |
| T1 coronal |  |  |  |  |  |
| Liver with contrast |  |  |  |  |  |
| Brain with contrast |  |  |  |  |  |
| T1FS post contrast (body) |  |  |  |  |  |

**Comment:** (optional)

**PRIMARY TUMOUR DETECTION**

Based on all available information, including ML output if available

Note: if a tumour crosses an anatomical boundary, please choose a single site of tumour (you can add a comment if you wish). If there are two separate primary tumours, you can add this as a second site.

|  | **1**  **No primary tumour identified at this site** | **2**  **Probably no primary tumour at this site** | **3**  **Probably primary tumour at this site** | **4**  **Highly likely primary tumour at this site** | **DM5 score**  **1-4**  **(NA if no ML output)** | RF5 used?  Please **tick** at any site where this was used for detection |
| --- | --- | --- | --- | --- | --- | --- |
| **RUL** |  |  |  |  |  |  |
| **RML** |  |  |  |  |  |  |
| **RLL** |  |  |  |  |  |  |
| **LUL** |  |  |  |  |  |  |
| **LLL** |  |  |  |  |  |  |
| **Max dimension (mm) if measurable primary tumour (NA if no primary tumour seen)** |  |  |  |  |  |  |

**Comment** (optional if second tumour or other comment):

**T stage (as per TNM version used in STREAMLINE study) based on all available information** If you identify a primary tumour at any site, with any confidence level, please tick one cell only; if no primary tumour identified at any site, please indicate. If two primary lesions are identified, then please stage according to the highest stage (you may add comment).

Note: if uncertain of stage then please select the most likely stage with corresponding level of confidence/uncertainty.

| *tick 1 box in this table | **1**  **very low confidence** | **2**  **low confidence** | **3**  **reasonable confidence** | **4**  **high confidence** |
| --- | --- | --- | --- | --- |
| **No primary tumour identified** |  |  |  |  |
| **T1** |  |  |  |  |
| **T2** |  |  |  |  |
| **T3** |  |  |  |  |
| **T4** |  |  |  |  |

**Comment:** (optional)

**REGIONAL NODES:**

Presence of nodal metastases based on all available information, including ML output if available.

| What is the regional nodal status based on all available information?  *-Please tick* | Negative  1- definitely not present  2- probably not present | | Positive/high confidence  3- probably present  4- highly likely present | | DM5 score  NA if no ML output | RF5 used?  Please tick at any site where this was used for detection |
| --- | --- | --- | --- | --- | --- | --- |
|  | **Negative** | | **Positive** | |  |  |
|  | 1 | 2 | 3 | 4 | 1-4 |  |
| Supraclavicular |  |  |  |  |  |  |
| Paratracheal |  |  |  |  |  |  |
| Pre-vascular |  |  |  |  |  |  |
| Right hilar |  |  |  |  |  |  |
| Left hilar |  |  |  |  |  |  |
| Subcarinal |  |  |  |  |  |  |
| Other regional nodal site (please describe or NA): |  |  |  |  |  |  |
| Other regional nodal site (please describe or NA): |  |  |  |  |  |  |

**Nodal stage (as per TNM version used in STREAMLINE study):**

Note: if uncertain of stage then please select the most likely stage with corresponding level of confidence/uncertainty

| *tick 1 box in this table | **1**  **very low confidence** | **2**  **low confidence** | **3**  **reasonable confidence** | **4**  **high confidence** | **DM5 stage (1-4)** | RF5 used?  Please tick at any site where this was used for detection |
| --- | --- | --- | --- | --- | --- | --- |
| **N0** |  |  |  |  |  |  |
| **N1** |  |  |  |  |  |  |
| **N2** |  |  |  |  |  |  |
| **N3** |  |  |  |  |  |  |

**Comment:** (optional)

**METASTASES: NON-SKELETAL SITES**

Based on all available information, including ML output if available. If “Negative 1 or 2” is selected by reader, no measurement required, even if ML score >2. If “Positive 3 or 4” is selected by reader, then size(s) should be given.

| Presence or absence of metastasis based on all available information  *-Please tick* | Negative  1- definitely not present  2- probably not present | | Positive  3- probably present  4- highly likely present | | Size of largest organ deposit *(mm)* | | Size of second largest organ deposit *(mm)*  *(if not applicable put N/A)* | | Number of additional deposits  ≥6mm  *(if ≤10, state number. If >10 state, >10)*  *(if not applicable put N/A)* | | Number of additional deposits  <6mm  *(if ≤10, state number. If >10 state, >10)*  *(if not applicable put N/A)* | | RF5 used?  Please tick at any site where this was used for detection |
| --- | --- | --- | --- | --- | --- | --- | --- | --- | --- | --- | --- | --- | --- |
|  | **Negative** | | **Positive** | |  |  |  |  |  |  |  |  |  |
|  | 1 | 2 | 3 | 4 |  |  |  |  |  |  |  |  |  |
|  |  |  |  |  |  | DM5 score 1-4 |  | DM5 score  1-4 |  | DM5  score  1-4 |  | DM5 score  1-4 |  |
| Brain |  |  |  |  |  |  |  |  |  |  |  |  |  |
| Lung (L) |  |  |  |  |  |  |  |  |  |  |  |  |  |
| Lung (R) |  |  |  |  |  |  |  |  |  |  |  |  |  |
| Pleura (L) |  |  |  |  |  |  |  |  |  |  |  |  |  |
| Pleura (R) |  |  |  |  |  |  |  |  |  |  |  |  |  |
| Liver (left lobe) |  |  |  |  |  |  |  |  |  |  |  |  |  |
| Liver (right lobe) |  |  |  |  |  |  |  |  |  |  |  |  |  |
| Spleen |  |  |  |  |  |  |  |  |  |  |  |  |  |
| Adrenal (L) |  |  |  |  |  |  |  |  |  |  |  |  |  |
| Adrenal (R) |  |  |  |  |  |  |  |  |  |  |  |  |  |
| Kidney (L) |  |  |  |  |  |  |  |  |  |  |  |  |  |
| Kidney (R) |  |  |  |  |  |  |  |  |  |  |  |  |  |
| Pancreas |  |  |  |  |  |  |  |  |  |  |  |  |  |
| Mesentery/peritoneum |  |  |  |  |  |  |  |  |  |  |  |  |  |
| Bowel |  |  |  |  |  |  |  |  |  |  |  |  |  |
| Soft tissue neck/chest |  |  |  |  |  |  |  |  |  |  |  |  |  |
| Soft tissue abdomen/pelvis |  |  |  |  |  |  |  |  |  |  |  |  |  |
| Nodal *(Non regional - Please state site; NA if no other nodal site)*________________ |  |  |  |  |  |  |  |  |  |  |  |  |  |
| Other *(Please state/ or NA if no other site)*________________ |  |  |  |  |  |  |  |  |  |  |  |  |  |

**Comment:** (optional)

**METASTASES: SKELETAL SITES**

Based on all available information, including ML output if available. If “Negative 1 or 2” is selected by reader, no measurement required, even if ML score >2. If “Positive 3 or 4” is selected by reader, then size(s) should be given.

| Presence or absence of metastasis based on all available information  *-Please tick* | Negative  1- definitely not present  2- probably not present | | Positive  3- probably present  4- highly likely present | | Size of largest organ deposit *(mm)* | | Size of second largest organ deposit *(mm)*  *(if not applicable put N/A)* | | Number of additional deposits  ≥6mm  *(if ≤10, state number. If >10 state, >10)*  *(if not applicable put N/A)* | | Number of additional deposits  <6mm  *(if ≤10, state number. If >10 state, >10)*  *(if not applicable put N/A)* | | RF5 used?  Please tick at any site where this was used for detection |
| --- | --- | --- | --- | --- | --- | --- | --- | --- | --- | --- | --- | --- | --- |
|  | **Negative** | | **Positive** | |  |  |  |  |  |  |  |  |  |
|  | 1 | 2 | 3 | 4 |  |  |  |  |  |  |  |  |  |
|  |  |  |  |  |  | DM5 score  1-4 |  | DM5 score  1-4 |  | DM5 score  1-4 |  | DM5 score  1-4 |  |
| Skull |  |  |  |  |  |  |  |  |  |  |  |  |  |
| Cervical spine |  |  |  |  |  |  |  |  |  |  |  |  |  |
| Thoracic spine |  |  |  |  |  |  |  |  |  |  |  |  |  |
| Lumbar spine |  |  |  |  |  |  |  |  |  |  |  |  |  |
| Pelvis |  |  |  |  |  |  |  |  |  |  |  |  |  |
| Sternum |  |  |  |  |  |  |  |  |  |  |  |  |  |
| Clavicle/Scapula (L) |  |  |  |  |  |  |  |  |  |  |  |  |  |
| Clavicle/Scapula (R) |  |  |  |  |  |  |  |  |  |  |  |  |  |
| Ribs (L) |  |  |  |  |  |  |  |  |  |  |  |  |  |
| Ribs (R) |  |  |  |  |  |  |  |  |  |  |  |  |  |
| Other *(Please State or NA)*    _________________ |  |  |  |  |  |  |  |  |  |  |  |  |  |

**Additional comments** (e.g. 2^nd^ primary, incidental benign findings) (optional)

Exam read end time: _________:_______ (24-hour clock, hours, minutes)

Now go back and fill in ML scores. Please tick.

Now go back to check table on page 2 is completed. Please tick.

CRF completed by (scribe): Signature:

CRF completed by (reader): Signature:

Date:
