# Supplementary material for: Development and Evaluation of Machine Learning in Whole-Body Magnetic Resonance Imaging for Detecting Metastases in Patients With Lung or Colon Cancer: A Diagnostic Test Accuracy Study
Source: Invest Radiol. 2023 Jun 26;58(12):823–31. doi: 10.1097/RLI.0000000000000996 (PMC10662596; doi:10.1097/RLI.0000000000000996)
Supplement: Supplementary file 4 [file ir-58-823-s004.docx]

#

# Statistical Analysis Plan (SAP) for Machine Learning In whole Body Oncology (XXXX) project (Version 1.1; 24th Jan 2020)

## Statistical analysis plan (SAP) Background

The use of WB-MRI for the detection of metastatic disease is an active area of research in oncology imaging. In particular diffusion weighted MRI (DW-MRI), which quantifies water diffusivity, can detect tumour sites in organs and bones. Limitations of WB DW-MRI are the risk of false positives (as many “normal” anatomical structures can look similar to pathological tissue) and the long reading times due to the large number of complex images. Machine learning techniques have previously been used to assist in reading MRI data by developing algorithms to differentiate between benign and malignant cases, though not using DWI. In this study machine learning methods using WB DW MRI will be developed and evaluated for staging of patients with cancer.

## Research questions

The primary research question is as follows. Is the specificity of WB DW-MRI scans, in patients being staged for cancer, significantly improved with no subsequent loss of sensitivity when machine learning methods are applied? The secondary research question includes:

1. Can the reading time (RT) of WB DW-MRI scans be reduced, with a reduction of associated radiology costs, when machine learning techniques are employed to assist experienced radiologists?
2. Can inter-observer variability be reduced by the use of machine learning methods in experienced or new WB-MRI radiologists?
3. Can the application of machine learning methods in WB-MRI increase the diagnostic accuracy delivered by less experienced radiologists?
4. Can intra-observer variability be reduced by the use of ML methods in experienced WB MRI radiologists (exploratory)?

## Study objective

The primary objective of this project is to compare the diagnostic accuracy of WB DW-MRI, as read by experienced radiologists, in patients being staged for cancer, with and without the aid of machine learning methods against the reference standard of full clinical diagnosis at 12 month follow-up period.

The secondary objectives are in the following:

1. To compare the reading time of WB-MRI scans
2. To assess inter-observer variability
3. To test the diagnostic accuracy of non-experienced radiologists

To achieve these objectives, we need to design statistical analysis experiments systematically.

## Design

- - 1. Patient data

This is an observational study using patient data from the STREAMLINE study. If deemed compatible with the developed algorithm, data from the MELT and MASTER studies may also be used.

The primary data source is STREAMLINE (8), a pair of multi-centre prospective cohort studies that evaluate WB-MRI in newly diagnosed non-small cell lung cancer (250 patients; STREAMLINE-L; ISRCTN50436483) and colorectal cancer (322 patients; STREAMLINE-C; ISRCTN43958015). The primary objective for both studies was to evaluate whether early WB-MRI increases detection rate for metastases compared to standard National Institute for Health and Care Excellence (NICE, <https://www.nice.org.uk/>)-approved diagnostic pathways. Secondary objectives included assessing influence of WB-MRI on time to and nature of first major treatment decision following definitive staging. At 12-month patient follow-up, a multidisciplinary consensus panel defined the reference standard for tumour stage considering all clinical, pathological, post mortem and imaging follow-up.

Cases from STREAMLINE are likely to have more non-nodal metastatic sites, such as liver and lytic bone metastases. Therefore, additional cases of nodal disease and sclerotic bone metastases will be acquired from two other studies to ensure variation in the distribution of disease used to develop the machine learning algorithm. The MELT study (100 patients; NCT01459224) is a prospective observational cohort study to compare staging accuracy using WB-MRI with standard investigations in patients with newly diagnosed Hodgkin's lymphoma. Data from the MASTER study, including cases with lymphoma and prostate cancer, will also be used (the myeloma cases from MASTER are unlikely to be used).

In addition, WB-MRI datasets from 51 healthy volunteers will be used; these have been collected under a separate ethics approval (08/H0707/58) (14).

- - 1. Study design

The statistical study design is mainly in the second and third phases of this project. The anatomical atlases from phase 1 was used in Phase 2 for anatomic mapping of healthy organs in the study scans.

In Phase 2, the machine learning produced a probabilistic map indicating the likelihood that the tissue is malignant. The scans and disease segmentations, based on the source study reference standard, was used to inform the algorithm using relatively sparse data. Training of the algorithm requires sufficient data from the three source studies to allow identification for different sites of disease, whilst holding back the data to be used in testing. The machine learning algorithm was refined in successive iterations until a final algorithm is obtained. An analysis of per lesion sensitivity was performed at this stage using approximately 40-50 new patient datasets (to allow for sufficient positive cases). If the upper 95% confidence interval (CI) of the sensitivity by algorithm ‘C’ is less than 80%, then further work on the algorithm will need to be undertaken prior to proceeding to Phase 3.

The assessment of study outcomes was carried out in the third phase of this project. A second set of WB-MRI data relating to 191 subjects from the STREAMLINE, MELT and MASTER studies was read by expert radiologists, both with and without ML support, in a similar way to a cross-over design. The timing and order of the reads was randomised. Study outcomes was recorded for each read. For the diagnostic accuracy measures, the “gold standard” was regarded as the reference standard from the main study. A subset of the scans was re-read (both with and without ML support) by another radiologist to assess inter-observer variability. A further subset was read by non-experienced radiologists to estimate diagnostic accuracy in this group.

- - 1. Groups for comparison

The groups for comparison was read with the aid of ML compared to reads without the aid of ML. The groups are paired as each scan will be read by the same radiologist with and without the aid of ML. The primary comparison was amongst expert radiologists and a separate, secondary comparison was amongst inexperienced radiologists for a subset of 30 patients.

## Study population

No patients were directly recruited into this study. Recruitment and scanning of patients have taken place under the separate contributing studies.

- - 1. Study population for contributing studies

**STREAMLINE L and C** study inclusion criteria: histopathologically confirmed or suspected lung cancer or colorectal cancer being staged for initial treatment planning; written informed consent. Exclusion criteria include any contra-indication to MRI scanning

**MELT (if applicable)** study inclusion criteria: aged 6-18 years with participant/guardian informed consent, histologically confirmed Hodgkin’s lymphoma, treated with the Euronet chemotherapy regime. Exclusion criteria: contra-indications to MRI, previous other malignancy or pregnancy/nursing

**MASTER (if applicable)** study inclusion criteria: diagnosis of prostate cancer, lymphoma or myeloma.

- - 1. Additional inclusion and exclusion criteria for this study

***Phase 1*** inclusion criteria: healthy volunteers aged 18-100 years, written informed consent.

Exclusion criteria: any co-existing medical illness, contra-indications to MRI.

***Phase 2*** inclusion criteria: patient eligible for and consented to take part in one of the contributing studies (STREAMLINE C/L plus, if applicable, MELT & MASTER), patient completed the study imaging assessments and the reference standard from the source study is available.

Exclusion criteria: Patients that consented to contributing study but did not undergo the scan, or the scan could not be adequately completed. In addition, cases will be excluded if the scan data is significantly corrupted during data transfer or contains significant artefacts, with marked obscuration of the images, such that the scan could not be reasonably processed for ML. This evaluation is made by the study MRI physicist and a record of each case will be maintained. Cases will also be excluded if the source study reference standard is not available.

***Phase 3*** inclusion criteria: any patients that were eligible for Phase 2 are automatically eligible for Phase 3.

Exclusion criteria: Any patients whose reads were used in Phase 2 for the development of the ML algorithm are not considered for Phase 3.

- - 1. Blinding

The study cannot be blinded to the addition of ML. The readers evaluating the sensitivity and specificity of WB-MRI with or without ML support will be blinded to the reference standard, including the original primary diagnosis and the stage of disease. To prevent any recall bias occurring in the Phase 3 analysis, readers will not be assigned scans originating from their home practice/site as part of the source studies.

- - 1. Sample size

The sample size required for phase 3 based on patients with no metastases is 141. This is based on McNemar’s test of paired proportions (McNemar, 1947), assuming ML support will improve specificity by 10%, from 86% to 96%, with a type 1 error of 0.05 (one-sided) and 90% power. Further As per section 9.1 of the protocol, it is expected that 193 cases from the STREAMLINE study are available for use. Amongst the 193 patients from STREAMLINE studies, 51 are expected to have metastatic disease. Based on the above, we are anticipating an improvement in specificity of 10% from 86%. Regarding sensitivity, on the assumption that the sensitivity of WB-MR with ML support is no less than that of WB-MR alone (88%), a sample size of 51 metastases will provide an expected 95% confidence interval for the sensitivity of WB-MR with ML support of 79% to 97%.

- - 1. Randomization

This study is not a randomized controlled trial, however stratified randomization will be employed to allocate scans between phases 2 and 3 to reduce the risk of a difference in the scans analysed in these phases. Randomisation will be stratified on: (i) type of primary tumour (colon, lung) (ii), presence of metastatic disease: liver, bone, nodal. Randomisation will be undertaken by the study statistician when complete reference standard data is available and the cases that could not be processed for ML have been determined by the physicist.

Randomisation will also be used to assign and order the cases. First, cases will be randomly assigned to readers, maintaining an equal number of cases per reader as far as possible, based on the strata of type of primary tumour, presence of metastatic disease and site such that each radiologist with have a similar proportion of each ‘type’. Once assigned to each reader, a random selection of half the cases will be chosen to be assigned ML first, and the remainder non-ML first. These scans will comprise the first set received by the reader. The reverse set will be provided once the first set has been completed and a month has elapsed.

In order to allow for the inter-reader analysis, each radiologist will be assigned 10 reads that will be read only by the assigned radiologist. The remainder will be read by two radiologists, randomly assigned such that the proportions of primary tumor type and presence of metastatic disease remain even amongst all expert radiologists.

In order to assess the effect of ML amongst non-experienced radiologists, the same randomisation procedure as detailed above for experienced radiologists will be utilised.

- - 1. Analysis Sets

The analysis population was included all WB-MRI scans where both the unassisted read and the ML-assisted read were completed by a radiologist.

## Variables of Analysis

- - 1. Primary outcome

The primary outcome measure was the per patient specificity of WB-MRI against the reference standard established in the main study. The observed data are summarized in Table 33:

Table 33. 2x2 table of observed per patient classification

(a) without ML and (b) with ML, against the reference standard. As the same scans are read both with and without ML, the marginal totals for the reference standard (n-, n+ and N) are the same in both (a) and (b).

|  | (a) | Patient classification without ML | | | (b) | Patient classification with ML | | |
| --- | --- | --- | --- | --- | --- | --- | --- | --- |
|  |  | Negative | Positive | Total |  | Negative | Positive | Total |
| Reference standard | Negative | a_1_ | b_1_ | a_1_+b_1_=n_-_ |  | a_2_ | b_2_ | a_2_+b_2_=n_-_ |
|  | Positive | c_1_ | d_1_ | c_1_+d_1_=n_+_ |  | c_2_ | d_2_ | c_2_+d_2_=n_+_ |
|  | Total | a_1_+c_1_ | b_1_+d_1_ | a_1_+b_1_+c_1_+d_1_=N |  | a_2_+c_2_ | b_2_+d_2_ | a_2_+b_2_+c_2_+d_2_=N |

Specificity is defined as the proportion of patients with negative reference standard (metastasis not present anywhere) which have been correctly classified as negative by the radiologist, i.e. a_1_/(a_1_+b_1_) without ML and a_2_/(a_2_+b_2_) with ML.

### Radiologist classification

WB DW-MRI will be assessed for the presence of disease, using an imaging volume from the brain to mid-thighs. Reads will proceed using all sequences from the source study, with experienced and non-experienced WB MRI readers. The sites of disease to be described will include the primary tumour site, presence and site of metastatic lesions and any significant incidental findings.

***Reference standard***

The reference standards for this study are taken from the contributing studies. For the STREAMLINE data, at 12-month patient follow-up, a multidisciplinary consensus panel defined the reference standard for tumour site and stage considering all clinical, pathological, post mortem and imaging follow-up. The reference standard for the MELT study is contemporaneous MDT with all other staging, e.g. PET CT and CT, at the time of diagnosis and initial staging.

- - 1. Secondary outcomes

***Per patient sensitivity*** of WB-MRI against the reference standard established in the main study. Sensitivity is defined as the proportion of patients with positive reference standard (at least one metastatic deposit) which have been correctly classified as positive by the radiologist, i.e. d_1_/(c_1_+d_1_) without ML and d_2_/(c_2_+d_2_) with ML.

***Per lesion specificity*** of WB-MRI against the reference standard established in the main study. Specificity is defined as the proportion of lesions with negative reference standard which have been correctly classified as negative by the radiologist. A similar table to Table 1 can be constructed on a per lesion basis.

***Per lesion sensitivity*** of WB-MRI against the reference standard established in the main study. Sensitivity is defined as the proportion of lesions with positive reference standard which have been correctly classified as positive by the radiologist.

***Confidence*** of the tumour detection diagnosis at site ‘x’ by the radiologist reading the WB MRI:

1. No primary tumour

Positive

Negative

1. Probably no tumour
2. Probably tumour present
3. Highly likely tumour present

Confidence of the T-Stage diagnosis by the radiologist reading the WB MRI:

1. very low confidence

Positive

Negative

1. low confidence
2. reasonable confidence
3. high confidence

***Tumour size:*** Size of the largest organ deposit, the second largest organ deposits, the number of additional deposits ≥ 6mm, the number of additional deposits < 6mm.

***Reading time:*** The total time taken by the radiologist to read and report the WB-MRI in minutes, not including time taken to complete the CRF.

***Inter-observer variability*** Inter-observer variability in a subset of scans with reads by two different radiologists will be measured by the kappa coefficient, generalised to non-unique raters (153) (154). Both ML and non-ML reads will be repeated. For each subject i, i=1,…,N, let x_i_ be the total number of positive diagnoses (0,1 or 2) from the two radiologists, and $\bar{p}$ be the overall proportion of positive ratings. Then the between-subject mean square error is approximated by:

$$B=\frac{1}{N}\sum_{i} \frac{{{(x}_{i}-2\bar{p})}^{2}}{2}$$

and the within-subject mean square error is:

$$W=\frac{1}{2N}\sum_{i} x_{i}(2-x_{i})$$

and kappa is defined as:

$$\kappa= \frac{B-W}{B+W}$$

Kappa is calculated separately for reads with and without ML assistance.

***Intra-observer variability*** will be measured and compared using the kappa coefficient in the same method as above. Instead of comparing ‘Reader 1’ and ‘Reader 2’, ‘Period 1’ and ‘Period 2’ will be used instead to represent the two different times the same read was assessed. Again, Kappa is calculated separately for reads with and without ML assistance and compared.

***Cost of radiology reading time*** measured as per hour staff costs in pounds sterling (GBP) for consultant radiologists.

- - 1. Other variables

The following variables are recorded both as part of the reference standard and the radiologist assessment in Phase 3.

- Site of tumour
- Tumour stage (N stage; M stage)

**Confidence** of the N/M-Stage diagnosis by the radiologist reading the WB MRI:

- very low confidence

Positive

Negative

- low confidence
- reasonable confidence
- high confidence
- Size of largest and second largest deposits at staging
- Number of additional deposits <6mm
- Number of additional deposits ≥6mm
- Radiologist ID
- Radiologist level (Experienced or Non-Experienced)
- Date of read

## Statistical Methodology

- - 1. General methodology

This SAP does not describe the ML aspect of the analysis. For clarity, the analysis described by this SAP is as follows:

- Interim analysis of the per lesion sensitivity in Phase 2
- Analysis of outcomes in Phase 3

Statistical significance is set at p=0.05 throughout and confidence intervals (CI). The primary outcome analysis comparing the per patient specificities with and without ML will be a one-sided test and confidence interval for the difference; all other tests and confidence intervals are two-sided. The only hypothesis test and p-value to be presented concerns the primary outcome and, as such, there will be no adjustment for multiple testing.

Results will be presented according to the STARD guidelines (155) where possible and checklist items have been noted in this SAP.

- - 1. Missing data (STARD item 16)

This study will use scans and follow-up data already collected within the STREAMLINE studies and also MELT and MASTER studies (if deemed compatible). Patients with missing or indeterminate reference standard data, or with missing or inadequate scan data from the source studies will not be eligible for this study, but numbers will be reported in the patient flow diagram. In phase 3, any missing data from the radiology reads will be queried with the reader and attempts made to complete the data. Any missing data remaining will be reported but excluded from the analysis. If only part of a patient’s report is missing, then the remainder of the data will be used where possible. Inconclusive diagnoses in phase 3 will be reported but excluded from the analysis.

As it is expected that missing data will be at a minimum, no data will be imputed for the purpose of the primary or secondary analysis.

- - 1. Baseline characteristics (STARD items 20 & 21)

The following information (from the reference standard) will be described for all cases, with those used in phase 2 and 3 shown separately:

- Location of primary tumour (colon, lung,)
- Maximum dimension of primary tumour (cm, median and interquartile range)
- Location of metastatic disease (liver, bone, nodal)
- N stage (N0, N1, N2, N3)
- M stage (M0, M1a, M1b)
  - 1. Primary outcome analysis (STARD item 23 & 24)

The per patient specificities of WB-MRI with and without ML, for expert radiologists, against reference standard was presented with 95% CI calculated using the Wilson method. The normal approximation is unsuitable as the proportions are likely to be close to 1. The proportions were compared using McNemar’s test for paired proportions (156). Using the same notation for the negative reference standard cases as used in Table 33 (a1, b1, a2, b2, n_-_), we can construct a 2x2 table to compare the (paired) proportions of patients classified as negative using the reference standard that are correctly identified as negative by the radiologist with and without ML (Table 34):

Table 34. 2x2 table to compare specificity with and without ML

|  |  | With ML | | |
| --- | --- | --- | --- | --- |
|  |  | Negative | Positive | Total |
| Without ML | Negative | I | J | a_1_ |
|  | Positive | K | L | b_1_ |
|  | Total | a_2_ | b_2_ | n_-_ |

The null hypothesis is that the two specificities (the marginal probabilities in Table 2) are the same, and the alternative hypothesis is that the specificity is higher with ML:

$$H_{0}:\frac{a_{1}}{n_{-}}=\frac{a_{2}}{n_{-}} \text{ }H_{1}:\frac{a_{1}}{n_{-}}>\frac{a_{2}}{n_{-}}$$

McNemar’s test statistic is:

$$T=\frac{{(j-k)}^{2}}{(j+k)}$$

Under the null hypothesis, T follows a χ_2_ distribution on 1 degree of freedom. The p-value from a one-sided test will be reported.

Results will be expressed as an absolute difference in proportions:

$$\Delta=\frac{a_{1}-a_{2}}{n_{-}}=\frac{j-k}{n_{-}}$$

As a one-sided test is being used, assuming the point estimate of the absolute difference in proportions is positive, the one-sided 95% CI is:

$$[\Delta-1.645\times SE\left( \Delta\right), +\infty]$$

where:

$$SE\left( \Delta\right)=\frac{1}{n_{-}}\sqrt{j+k- \frac{{(j-k)}^{2}}{n_{-}}}$$

A one-sided test is being used as it is not expected that specificity could be worsened by the addition of ML assistance. In the event that the point estimate is negative, a two-sided test and CI will be presented, acknowledging the loss in power to demonstrate a difference.

Should the number of discordant pairs (j+k) be small, an exact test will be performed instead. The p-value is calculated from the binomial distribution as:

$$\min\left\{ 1, \sum_{t=0}^{\text{min(j,k)}} \left( \begin{matrix} j+k \\ t \end{matrix} \right)\left( \frac{1}{2} \right)^{j+k} \right\}$$

- - 1. Secondary outcome analysis

**Per patient sensitivity, per lesion sensitivity and per lesion specificity** of WB-MRI with and without ML, for expert radiologists, against the reference standard were reported with 95% confidence intervals. The difference in sensitivity/specificity and 95% confidence interval were calculated similarly to the primary outcome though no hypothesis test was performed.

**Per patient sensitivities and specificities** of WB-MRI with and without ML, for inexperienced radiologists, against reference standard was reported with 95% confidence intervals. The difference in sensitivity/specificity and 95% confidence interval was calculated similarly to the primary outcome though no hypothesis test will be performed.

**Confidence:** The agreement in confidence (on a scale of 1 to 4) between WB MRI with and without the ML support will be described using a 4x4 table, as below, and visualised using a bar charts. This will be done overall and by reference standard diagnosis.

Table 35. Comparison of confidence in diagnosis

|  |  | | Confidence with ML | | |
| --- | --- | --- | --- | --- | --- |
|  |  | | 1 | … | 4 |
| Confidence without ML | | 1 |  |  |  |
|  |  | … |  |  |  |
|  |  | 4 |  |  |  |

**Tumour size** as assessed with and without ML will be compared using scatter plots. The size of the largest deposit as measured with ML will be plotted against the size of the largest deposit as measured without ML. A reference line of x=y will be added to indicate the same size measured by both methods, and colours used to indicate the reference standard diagnosis. This will be repeated for the size of second largest deposit, and the number of additional deposits.

**Reading time** (RT) will be compared between WB MRI with and without the ML support by calculating the paired difference (RT with ML – RT without ML) for each scan, as they are read by the same radiologist. The paired differences will be analysed using regression, adjusting the standard errors for clustering at the radiologist level and including covariates: order of reads (ML first/second) and type of primary tumour (colon, lung). The estimated mean difference in RT (with 95% confidence interval) at the mean level of covariates will be obtained from the intercept term in the regression. The associations with read order and type of tumour will also be reported. The regression is carried out on the assumption that all readers have a one-month gap between reading sessions under the provision that the gap is sufficient enough to remove any recall bias. In the event where times between reading sessions vary between readers, an additional covariate may be added and its interaction with other covariates to explore dilution of their effects due to recall (time between reads in days).

A transformation of the dependent variable may be required if the regression assumptions are not met. If the assumptions are still not met after transformation, a Wilcoxon signed rank test (157) was used to test for an unadjusted difference in RT.

**Inter-observer variance**: Summary statistics of the proportions of concordant and discordant diagnosis between two experienced radiologists will be reported for both methods. Inter-observer variance will be measured by kappa (κ) coefficient as described in section 6.2. 95% confidence intervals for kappa will be calculated using bootstrapping (bias-corrected method). We will assess whether the inter-observer variance is reduced using ML by comparing the estimated values of kappa with and without ML, using the method of Gwet (158) which is similar to the paired t-test.

Intra-observer variance: this will be calculated and compared using the same methodology as above replacing the reads from two different experienced radiologists with tow reads from the same radiologist, taken at a different time.

**Costs of radiology reading time:** Estimated cost savings per WB-MRI will be calculated by multiplying any reduction in reading time in hours (as per the above RT analysis) by the associated hourly radiologist reading costs. If appropriate, this will be performed separately for cases with and without metastases.

- - 1. Analysis for Non-Experienced Readers

In addition to the above analyses described in sections A.7.4 & A.7.5, to satisfy the third secondary objective, a duplicate set of analyses will be carried out based on reads carried out by a cohort of approximately 7-8 non-experienced readers. Likewise, difference in ML-effect sizes between experienced and non-experienced cohorts will be assessed to investigate whether any effect derived from using ML output is affected by the experience of reader.

The non-experienced reader cohort may potentially have a variance in abilities (for example, some may be at consultant level with experience of reporting cancer whilst some are at the trainee level). In the event where this occurs, an additional set of sensitivity analyses will be run allowing for the inclusion of a stratification variable for ‘ability’ (consultants/trainees/etc).

- - 1. Subgroup analysis

The following subgroup analysis was performed for diagnostic accuracy outcomes, with subgroups defined by the reference standard:

- Size of lesion (above or below median)
- Location of primary tumour (colon, lung)
- Location of metastatic disease (liver, bone, nodal)
- N stage
- M stage
  - 1. Interim analysis

Interim analysis concerning per lesion sensitivity will be undertaken as part of Phase 2. It will be carried out using 40-50 new patient datasets. The per lesion sensitivity will be calculated as the proportion of metastatic lesions which are correctly identified by the ML algorithm. Correct identification is defined as achieving a particular threshold for the DICE co-efficient, which quantifies the overlap of the areas identified as lesions by the ML algorithm with the true lesion area, as defined by the clinical expert. A suitable threshold for the DICE coefficient will be defined as part of the ML process, prior to the interim analysis. A 95% confidence interval for the sensitivity will be calculated using the Wilson method (159). We will require the upper 95% CI of the sensitivity no less than 80%. If this is not met, then further work on the algorithm will be required. This is not a formal stopping rule, but rather the check to prevent proceeding to phase 3 if the algorithm is not identifying lesions at all. The cases used for the first interim analysis will not have been used for ML training or read by radiologists. Therefore, if the algorithm is sufficiently sensitive to proceed to phase 3, the cases used in the first interim analysis can be part of the validation set. They will therefore be selected from those allocated to phase 3.

A proposed interim analysis for per patient specificity for reads assisted by the ML algorithm in Phase 3 will no longer be performed due to time constraints.

- - 1. Sensitivity analysis

Any difference in specificity seen in the primary analysis may be affected by the order or timing of the scans. It is also possible that the statistical significance is overstated if outcomes for the same radiologist are correlated. Conditional logistic regression will be used to obtain an odds ratio comparing the specificity with and without ML adjusting for the scan order (ML first/second), time between scans (if regression assumptions in Section A.7.5 do not hold), and using robust standard errors to allow for clustering by radiologist.

To investigate whether the quality of sequence has an effect on the study results the primary analysis will be re-run using just reads where either the T2 Axial Stack or the DW Axial Stack has been deemed ‘good’ quality. The same set of reads will be used to re-run secondary analyses for per patient specificity and sensitivity. To investigate the effect sequence quality on read time, the regression model in Section A.7.5 will include covariates for T2 stack quality and DW stack quality.

As per Section A.7.6, in the event where the non-experienced reader cohort has varying grades of ability/experience. An additional sensitivity analysis will be run within the non-experienced cohort, including an additional variable to consider any effect this ability difference may have.

## Presentation of results

In addition to the tables and graphs described in sections A.7.4 and A.7.5, the following tables were presented.

Table 36. Diagnostic accuracy measures with and without ML assistance, read by experienced radiologists.

A similar table will be produced for diagnosis by inexperienced radiologists and for subgroup analyses.

|  | Without ML | With ML | Absolute difference  % (95% CI)^*^ |
| --- | --- | --- | --- |
| Per patient specificity, n/N % (95% CI) |  |  |  |
| Per patient sensitivity, n/N % (95% CI) |  |  |  |
| Per lesion specificity, n/N % (95% CI) |  |  |  |
| Per lesion sensitivity, n/N % (95% CI) |  |  |  |

Table 37. Summary table for secondary outcomes

|  | Experienced radiologists | | Inexperienced radiologists | |
| --- | --- | --- | --- | --- |
|  | Without ML | With ML | Without ML | With ML |
| Confidence, median (IQR) |  |  |  |  |
| Reference positive |  |  |  |  |
| Reference negative |  |  |  |  |
| Reading time, median (IQR) |  |  |  |  |
| Inter-observer variance, κ (95% CI) |  |  |  |  |
| Intra-observer variance, κ (95% CI) |  |  |  |  |
