# Supplementary material for: Development and Evaluation of Machine Learning in Whole-Body Magnetic Resonance Imaging for Detecting Metastases in Patients With Lung or Colon Cancer: A Diagnostic Test Accuracy Study
Source: Invest Radiol. 2023 Jun 26;58(12):823–31. doi: 10.1097/RLI.0000000000000996 (PMC10662596; doi:10.1097/RLI.0000000000000996)
Supplement: Supplementary file 5 [file ir-58-823-s005.docx]

**Supplemental Digital Content 1.**

**Sites of disease identified from the reference standard for ground truth segmentation**

**Sites of disease identified on the test set (n=188 patients) and associated ground truth segmentations**

Sites of disease for test set based on consensus reference standard and ground truth (GT) segmentation files (visible sites of disease).

**Supplemental digital content table S1:** Summary table displaying the number of cases from the consensus reference standard from which GT segmentations were created by each lesion type. The frequency of each metastatic site in the cohort is also reported as a percentage as well as the number of T2w and DW segmentation files created. N1-3 = nodal stage of disease, RL = right lobe, LL = left lobe, R = right, L= left, both = cases in which metastases were present on both the right and left sides of an organ.
