# Supplementary material for: Development and Evaluation of Machine Learning in Whole-Body Magnetic Resonance Imaging for Detecting Metastases in Patients With Lung or Colon Cancer: A Diagnostic Test Accuracy Study
Source: Invest Radiol. 2023 Jun 26;58(12):823–31. doi: 10.1097/RLI.0000000000000996 (PMC10662596; doi:10.1097/RLI.0000000000000996)

**Supplemental Digital Content 2.**

1. **Machine learning model development**

We initially investigated different machine learning algorithms for the task of malignancy lesion segmentation in WB-MRI scans. We explored classification forests (CFs) and deep convolutional neural networks (CNNs), as these had been used in other WB-MRI segmentation problems (see ref 11). For CFs, we use offset box features calculating intensity averages within randomly sized and displaced 3D boxes. Like Lavdas et al. (11), we use single-box and two-box features. Single-box features correspond to the average intensity of all voxels from a particular MRI channel within a randomized 3D box. Two-box features calculate the intensity difference between the averages in two randomized boxes. For two box features, each box intensity response is randomly taken from a different MRI channel. We used 100 trees with a maximum tree depth of 30. The stopping criterion is if either the objective function (information gain) cannot be further improved or the number of training samples in a leaf fall below a threshold of ten samples. A more detailed explanation of CFs can be found in reference 11. An open-source implementation of the used CFs is available at <https://github.com/biomedia-mira/oak2>. For the CNN model, we use the DeepMedic architecture (15) with dual pathway processing of input images at different resolution levels. This allows features to capture both local and contextual information, which is beneficial for whole body image segmentation. We use the default configuration for DeepMedic corresponding to an 11-layer deep CNN, where the last two layers correspond to fully connected layers. We use 50 feature maps in each layer. An open-source implementation of DeepMedic is available at https://github.com/deepmedic/deepmedic.

Our first experiments were conducted in a setting where an ML model was trained directly for the binary lesion segmentation using the manual annotations for the phase 2 training data. This resulted in binary classification methods that assigned a probability to each voxel in a multi-channel WB-MRI. Higher probabilities indicated that a voxel was more likely to be part of a malignant lesion. Because of the large volume of the WB-MRI scans and the relatively small volume of annotated malignant lesions, there was a substantial imbalance between normal and malignant voxels (from the Streamline reference standard data: of the largest non-skeletal largest metastases, the mean diameter was 23.7mm, median diameter was 15mm, with a range of 2mm to 110mm). This affected the performance of this one-stage approach resulting in models with low sensitivity and was deemed insufficient for the purpose of the study. We also observed that CFs underperformed compared to CNNs, which was in line with the results reported in (11).

We therefore developed a two-stage approach for malignant lesion detection, repurposing an algorithm for segmentation of healthy organs that was developed in a previous healthy volunteer study (9). The organ masks were then mapped back with the inverse transformation to the original patient training data. For the patient training dataset, there was no reference segmentation of organs to compare with, so we assessed the quality of these segmentations visually and they appeared to be sufficient for the following purpose. The automatically generated organ maps were then merged with the manually segmented primary and metastatic malignant lesions on T2WI and DWI training data (see Figure 1 in main text). This resulted in all scans allocated to training having multi-class segmentation maps where the organ labels were generated automatically using the previously developed CNN algorithm, while the cancer lesions were labelled manually. We then used the training set for training the two multi-class algorithms (CFs and CNNS) which are both capable of predicting jointly the organ labels and malignant lesions. This multi-class approach resulted in a much better distribution of voxels over the class labels, with an easier task to learn class-specific features to develop the ML algorithm, rather than the binary task where all normal structures are merged into a single class. We then used the internal validation set (n=45) to test the performance using recall, precision and Dice scores. The results of are summarized in Figure S1. The curves demonstrate that the area under the Dice and precision curves was highest for the two-stage CNN model using DeepMedic (Figure S1-A), followed by the two-stage CFs (Figure S1-B). The two-stage approaches performed better than a single stage binary lesion segmentation model (Figure S1-C). The best performing model, the two-stage CNN, which was also preferred by expert readers on inspection of the visual output, was selected for the use with human readers.

**Figure S1**

**Recall, precision and Dice scores of model performance on internal validation dataset (n=45)**

A: DeepMedic _multiclass. Multiclass refers to the ability of the algorithm to simultaneously predict organ labels and lesions. DeepMedic (DM) is a convolutional neural network (CNN) based algorithm. This graph demonstrates higher area under the curve for Dice and precision compared to graphs B and C.

B. Random Forest_multiclass. This model included the multiclass approach but used Random Forest instead of a CNN.

C. DeepMedic_binary curve. In this case, the model is trained only with the lesion annotations, without initial organ segmentation (the one step approach). The plot demonstrates a worse performance compared to the DM multiclass approach shown in graph A with significantly lower area under the curve for recall, precision and Dice.

D. Panel of the three curves for comparison side-by-side.

S1 (A) DeepMedic_multiclass performance curves


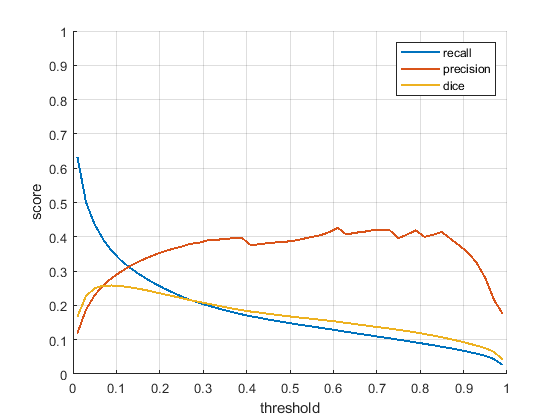


Figure S1 (B). RF_multiclass performance curves


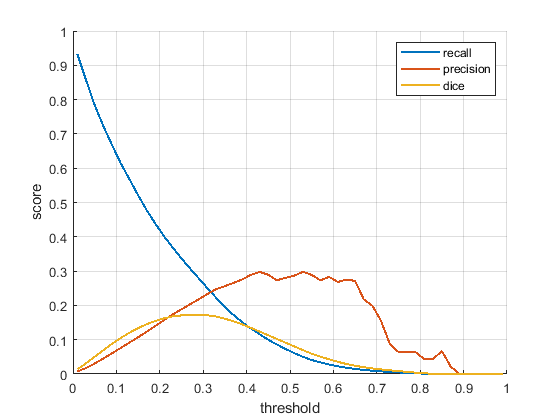


Figure S1 (C) DeepMedic_binary performance curves


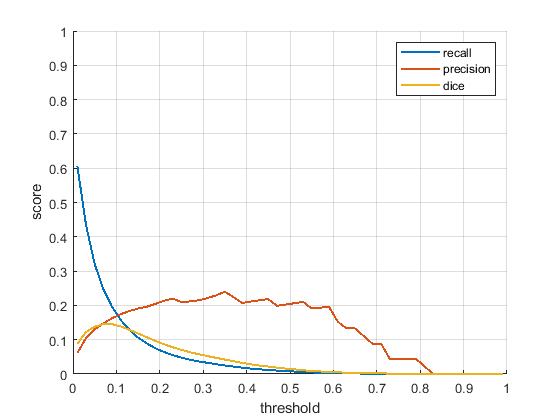


Figure S1 (D) Panel demonstrating the curves side-by-side.


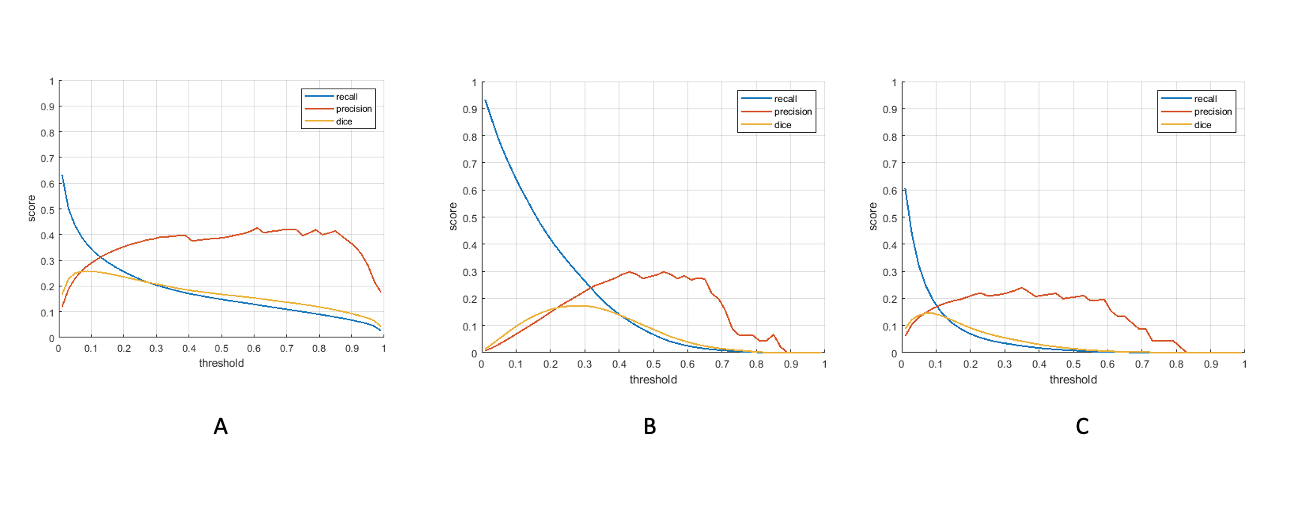

Supplement: Supplementary file 6 [file ir-58-823-s006.docx]
